# Acute Blood Pressure Response to Different Types of Isometric Exercise: A Systematic Review with Meta-Analysis

**DOI:** 10.31083/j.rcm2402060

**Published:** 2023-02-10

**Authors:** Juliana C. CONEGLIAN, Guilherme T. BARCELOS, Antonio Cleilson N. BANDEIRA, Ana Carolina A. CARVALHO, Marilia A. CORREIA, Breno Q. FARAH, Raphael M. RITTI-DIAS, Aline M. GERAGE

**Affiliations:** ^1^Post-graduate Program in Physical Education, Federal University of Santa Catarina, 88040-001 Florianópolis (SC), Brazil; ^2^Sports Center, Federal University of Santa Catarina, 88040-001 Florianópolis (SC), Brazil; ^3^Post-graduate Program in Medicine, Universidade Nove de Julho, 01525-000 São Paulo (SP), Brazil; ^4^Post-graduate Program in Physical Education, Federal University of Pernambuco, 52171-900 Recife (PE), Brazil; ^5^Post-graduate Program in Rehabilitation Sciences, Universidade Nove de Julho, 01525-000 São Paulo (SP), Brazil

**Keywords:** physical exercise, acute pressure response, cardiovascular safety

## Abstract

**Background:**

This study aimed to identify the blood pressure (BP) responses during different types of isometric exercises (IE) in adults and to evaluate whether BP responses according to IE is influenced by the characteristics of participants and exercise protocols.

**Methods:**

The search was conducted in PubMed, Cochrane Central, SPORTDiscus, and LILACS databases in June 2020. Random effects models with a 95% confidence interval and *p *< 0.05 were used in the analyses.

**Results:**

Initially, 3201 articles were found and, finally, 102 studies were included in this systematic review, seven of which were included in the meta-analysis comparing handgrip to other IE. Two-knee extension and deadlift promoted greater increases in systolic (+9.8 mmHg; *p* = 0.017; $I^{2}$ = 74.5% and +26.8 mmHg; *p *≤ 0.001; $I^{2}$ = 0%, respectively) and diastolic (+7.9 mmHg; *p* = 0.022; $I^{2}$ = 68.6% and +12.4 mmHg; *p *≤ 0.001; $I^{2}$ = 36.3%, respectively) BP compared to handgrip. Men, middle-aged/elderly adults, hypertensive individuals, and protocols with higher intensities potentiate the BP responses to handgrip exercise (*p *≤ 0.001).

**Conclusions:**

IE involving larger muscle groups elicit greater BP responses than those involving smaller muscle masses, especially in men, middle-aged/elderly adults and hypertensive individuals. Future studies should directly compare BP responses during various types of IE in different populations.

## 1. Introduction 

Handgrip strength has been considered a marker of general strength due to positive association with lower limb strength [1] and also has been associated with several health outcomes as mortality [2] health-related quality of life [3] and cognitive performance [4] in clinical populations. In addition, it has been used as an indicator of muscle strength in intervention studies in different populations [5, 6].

Otherwise, the isometric handgrip training has been used to improve cardiovascular health [7, 8, 9], given the reduction in blood pressure (BP) and improvement in endothelial function after a few weeks of intervention. The most commonly used handgrip protocol consists of four two-minute sets of contractions at 30% of maximal voluntary contraction (MVC) with a recovery interval of one to four minutes [9, 10, 11]. This modality of exercise appears to be safe from a cardiovascular point of view [12, 13], but there is no clarity about the magnitude of BP increase identified during its performance.

In addition, lower limb isometric exercises, involving larger muscle masses, have also been shown to be effective for chronic BP reduction [14, 15, 16]. However, the BP responses during these modalities of isometric exercise (IE) are unclear. Therefore, there are no recommendations for their adoption as a safe antihypertensive strategy.

Regarding the characteristics of the exercise protocol, greater muscle mass [17, 18], intensity [19, 20], frequency, and duration of contraction [21] seem to promote greater increases on the BP response during dynamic strength exercise. However, the influence of these factors on BP responses to IE still needs to be confirmed.

Moreover, the influence of subjects’ personal characteristics on acute BP responses to IE also needs to be investigated, trying to identify which groups of subjects would be at increased risk of acute events. Some studies show that men and older individuals present greater BP responses to IE compared to their pairs [22, 23, 17] while others have observed no difference [18].

Although isometric handgrip has recently been included as a complementary non-pharmacological strategy for the prevention and treatment of hypertension [24, 25, 26], there is still reluctance by international organizations to add this exercise modality in exercise guidelines to the same extent as dynamic resistance exercise [13, 27], since its cardiovascular safety is not yet well established, especially considering other exercises involving larger muscle mass.

In this context, to the best of our knowledge, there are no review studies that evidence the BP responses during the execution of different types of IE in adults. Thus, this systematic review with meta-analysis aimed to identify the BP responses during different types of IE in isolation and compared to handgrip in adults, and to identify such responses according to the characteristics of participants and exercise protocols.

## 2. Materials and Methods

This study protocol was previously registered with PROSPERO (CRD42020190823) and followed PRISMA guidelines [28].

### 2.1 Eligibility Criteria for Studies

Studies with any experimental design (randomized or not and controlled or not) were included, respecting the eligibility criteria established according to the acronym PICO (Population, Intervention, Comparator, and Outcome) [28]. Inclusion criteria were: adult participants (≥18 years), hypertensive or normotensive of both sexes, trained and untrained; IE of any type, intensity, volume, and load control; presence or absence of a comparator group with another type of IE; relating systolic blood pressure (SBP) and/or diastolic blood pressure (DBP) values, assessed before and during exercise or the difference between the two moments (delta). Furthermore, studies in Portuguese, English, or Spanish, available in full and published in any year were included.

Exclusion criteria were: adults with any comorbidity (except hypertension) or specific condition (e.g., pregnant women); studies with other interventions associated with IE; investigating the effects of medications; with IE performed after or randomly with other exercise modalities; that performed several stress tests on the same day before IE (without randomization), and with incremental testing; comparing IE with another exercise modality, without having a separate group for IE; with SBP and/or DBP measurements only after the exercise and only mean BP data.

### 2.2 Search Methods for Identification of Studies

The search for articles was conducted in the PubMed, Cochrane Central, SPORTDiscus, and LILACS databases in the month of June 2020. The search strategy, used for all databases, is available in **Supplementary Material 1**.

### 2.3 Study Selection and Data Extraction

The EndNote® X9.3.3 software (Philadelphia, PA, USA) was used to manage references and remove duplicates. First, the selection of articles was based on title and abstract reading by two independent researchers (GTB. and JCC.). The next step consisted of reading the full texts and selecting the studies according to eligibility criteria. In both steps, if there were disagreements between researchers, a third researcher (AMG) was consulted to reach a consensus.

Data extraction was performed by the same researchers, in a standardized and independent way. The following information regarding the participants was extracted: number of participants, percentage of women in the sample, age, ethnicity/race, training status, body mass, body mass index (BMI), and BP level classification. For the BP level classification, we considered the report of each study and not the resting BP value. If the study did not clearly report this information, we considered it as “not reported”. For the exercise protocol, it was considered: number and duration of sets, interval between sets, and intensity of effort. Regarding the outcome of the studies, the following were considered: SBP and DBP before (rest measurement) and during exercise or the difference between the two moments (delta), with mean and dispersion measures.

### 2.4 Risk of Bias Assessment

The risk of bias analysis was feasible only for the studies that compared handgrip with other IE, due to the various types of study designs included in this systematic review. In this case, the risk of bias was assessed by the same researchers who screened the studies and extracted the data, according to the Cochrane Handbook for Systematic Reviews of Interventions [29], considering random sequence generation, allocation concealment, blinding of participants and professionals, blinding of outcome assessors, incomplete outcomes, selective outcome reporting, and BP measurement method (other bias). It was classified as high, unclear or low risk [30]. Also, the criteria were classified as not applicable when it was not possible to be assessed due to the study design.

### 2.5 Data Analysis

All descriptive data are presented as mean and standard deviation (SD). Delta values for BP were calculated (BP during exercise - baseline BP). The overall effect for each type of exercise and the subgroup analyses were calculated from the mean difference between the pre-exercise BP and the BP during exercise. The comparison of BP between the IE types was performed using the mean values for each exercise type. Also, the effect of the comparison between the handgrip exercise and other exercise types was calculated from the mean difference in BP change between them. The SD of change was calculated from the pre-exercise and during-exercise SD values, adopting a correlation coefficient of 0.5. Meta-analyses were calculated using random effects models. Statistical heterogeneity between studies was assessed by the $I^{2}$ inconsistency test; considering that values above 50% indicate high heterogeneity [29]. Forest plots were generated to represent the combined effect and standardized mean differences with 95% confidence interval (CI), and *p*-values < 0.05 were considered statistically significant. The analyses were performed using Comprehensive Meta Analysis software version 2.2.064 (Englewood, NJ, USA.).

## 3. Results

### 3.1 Search Results

**Fig. 1.**
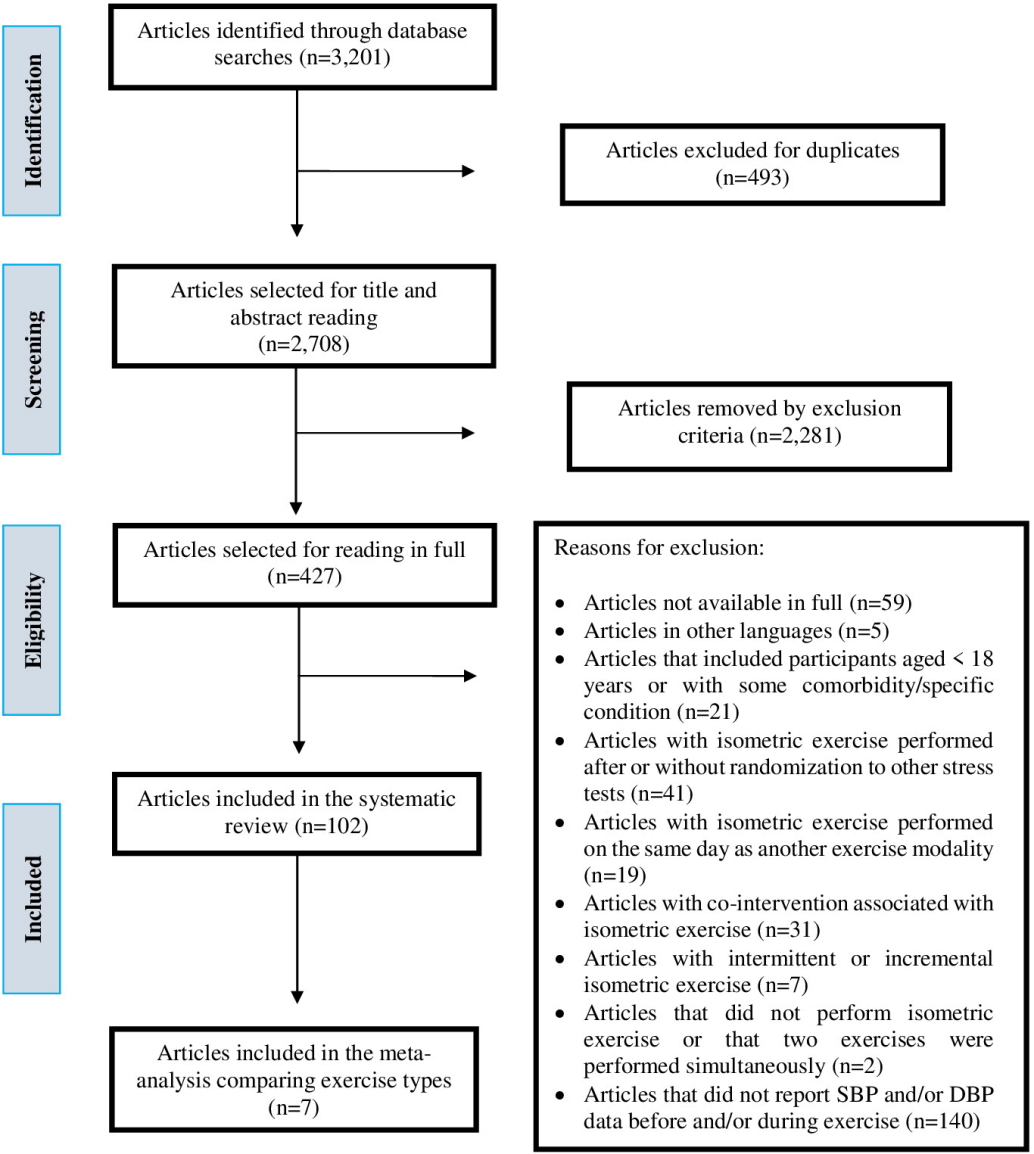
**Flowchart of the different steps of the systematic review**.

Initially, 3201 articles were found (Pubmed = 2170, Cochrane = 381, Lilacs = 237, and SPORTDiscus = 413) and 102 studies were, finally, included in the systematic review. Of these, seven were included in the meta-analysis comparing handgrip with others IE (Fig. 1).

### 3.2 Characteristics of the Studies

In summary, the studies of this systematic review included 12 types of IE and some of them evaluated more than one type of IE. Among these studies, the vast majority (76.5%) performed the handgrip, followed by knee extension (13.7%) (Table 1, Ref. [8, 13, 18, 19, 20, 22, 23, 31, 32, 33, 34, 35, 36, 37, 38, 39, 40, 41, 42, 43, 44, 45, 46, 47, 48, 49, 50, 51, 52, 53, 54, 55, 56, 57, 58, 59, 60, 61, 62, 63, 64, 65, 66, 67, 68, 69, 70, 71, 72, 73, 74, 75, 76, 77, 78, 79, 80, 81, 82, 83, 84, 85, 86, 87, 88, 89, 90, 91, 92, 93, 94, 95, 96, 97, 98, 99, 100, 101, 102, 103, 104, 105, 106, 107, 108, 109, 110, 111, 112, 113, 114, 115, 116, 117, 118, 119, 120, 121, 122, 123, 124, 125]).

**Table 1.** **Characteristics of the studies**.

Author and year	Country of origin	Modality	Sample (%women)	Age (years)	Ethnicity/race	Trainability status	Body mass (kg)	BMI (kg/$m^{2}$)	BP level classification
Almeida *et al*. (2021) [8]	Brazil	Handgrip	14.0 (79.0%)	24.5 ± 3.7	NR	Sedentary	NR	22.8 ± 2.2	Normotensive
			14.0 (50.0%)	26.6 ± 5.6				24.2 ± 3.7	
Aoki *et al*. (1983) [31]	Japan	Handgrip	18.0 (0.0%)	38.7 ± 3.2	Japanese	NR	64.1 ± 5.7	NR	Normotensive
			50.0 (0.0%)	40.5 ± 4.0			61.6 ± 7.1		Hypertensive
Auerbach *et al*. (2000) [32]	Israel	Whole-body isometric exercise	18.0 (0.0%)	51.1 ± 4.0	NR	NR	80.5 ± 11.0	NR	Normotensive
Bakke *et al*. (2007) [33]	Norway	Handgrip	11.0 (64.0%)	24.2 ± 5.1	NR	NR	66.6 ± 10.0	23.3 ± 2.0	Normotensive
			11.0 (36.0%)	62.7 ± 3.3			77.4 ± 9.9	25.0 ± 2.2	
Bakke *et al*. (2009) [34]	Norway	Handgrip	9.0 (33.0%)	23.6 ± 2.1	NR	NR	68.0 ± 10.2	22.5 ± 1.8	Normotensive
Balmain *et al*. (2016) [35]	Australia	Handgrip	19.0 (0.0%)	23.0 ± 2.0	NR	NR	70.9 ± 5.0	NR	Normotensive
Ben-Ari *et al*. (1992) [36]	Israel	Two-hand pulling	25.0 (0.0%)	47.0 ± 4.0	NR	Untrained	NR	NR	Normotensive
Bentley and Thomas (2018) [37]	Canada	Handgrip	20.0 (100.0%)	57.7 ± 5.2	NR	Moderately active	NR	26.9 ± 3.7	Normotensive
Borghi *et al*. (1988) [38]	Italy	Handgrip	16.0 (37.5%)	NR	NR	NR	NR	NR	Normotensive
Bosisio *et al*. (1980) [39]	Italy	Handgrip	8.0 (0.0%)	NR	NR	Trained (non-athlete)	NR	NR	Normotensive
Cottone *et al*. (1998) [40]	Italy	Handgrip	12.0 (42.0%)	38.0 ± 6.0	NR	NR	NR	25.7 ± 1.7	Normotensive
			15.0 (47.0%)	43.0 ± 3.0				26.0 ± 3.9	Hypertensive
Davies and Starkie (1985) [41]	England	Elbow flexion	11.0 (0.0%)	21.5 ± 7.3	NR	NR	NR	NR	Normotensive
		Plantar flexion							
Da Silva *et al*. (2013) [20]	Brazil	Leg press (45°)	AI: 8.0 (0.0%)	30.6 ± 6.2	NR	Physically active	74.4 ± 8.6	24.7 ± 2.6	Normotensive
			MI: 8.0 (0.0%)	31.6 ± 6.6			72.3 ± 13.9	24.2 ± 2.7	
			BI: 8.0 (0.0%)	27.5 ± 4.6			74.2 ± 15.8	25.5 ± 3.1	
Dias and Polito (2015) [42]	Brazil	Squat	19.0 (53.0%)	26.8 ± 7.3	NR	Sedentary	72.3 ± 14.9	24.7 ± 3.4	Normotensive
Ehsani *et al*. (1981) [43]	United States	Handgrip	14.0 (14.0%)	NR	NR	NR	NR	NR	Normotensive
Ehsani *et al*. (1982) [44]	United States	Handgrip	12.0 (8.0%)	NR	NR	NR	NR	NR	Normotensive
Ferguson and Brown (1997) [45]	England	Handgrip	5.0 (0.0%)	22.0 ± 1.8	NR	Athlete	NR	NR	NR
			10.0 (0.0%)	20.0 ± 4.7		Sedentary			
Fu *et al*. (1981) [46]	Japan	Handgrip	20.0 (NR)	54.9 ± 6.3	NR	NR	NR	NR	Normotensive
			35.0 (34.0%)	56.3 ± 9.5					Hypertensive
Fu *et al*. (2002) [47]	United States	Handgrip	5.0 (0.0%)	41.0 ± 2.2	NR	NR	84.0 ± 13.4	NR	Normotensive
Fujisawa *et al*. (1996) [48]	Japan	One-knee extension	7.0 (0.0%)	24.0 ± 3.0	NR	NR	63.9 ± 17.3	NR	Normotensive
Gois *et al*. (2020) [49]	Brazil	Handgrip	15.0 (NR)	53.0 ± 5.0	NR	Insufficiently active	75.0 ± 15.0	25.0 ± 3.0	Normotensive
Goldstein and Shapiro (1988) [50]	United States	Handgrip	20.0 (0.0%)	20.4 ± 3.2	NR	NR	NR	NR	Normotensive
Goldstraw and Warren (1985) [51]	England	Handgrip	12.0 (NR)	30.0 ± NR	NR	NR	NR	NR	NR
			12.0 (NR)	73.0 ± NR					
Goulopoulou *et al*. (2010) [52]	United States	Handgrip	23.0 (43.5%)	22.0 ± 1.4	NR	Physically active	76.9 ± 17.7	26.0 ± 4.8	Normotensive
Graafsma *et al*. (1989) [53]	Netherlands	Handgrip	10.0 (50.0%)	42.6 ± 9.1	NR	NR	NR	23.3 ± 2.9	Normotensive
			13.0 (46.0%)	39.1 ± 10.4				24.1 ± 3.0	Hypertensive
Greaney *et al*. (2013) [18]	United States	Handgrip	10.0 (0.0%)	24.0 ± 3.2	NR	NR	75.0 ± 9.5	23.2 ± 1.9	Normotensive
			9.0 (0.0%)	59.0 ± 6.0			87.0 ± 6.0	28.5 ± 3.9	
Greaney *et al*. (2014) [54]	United States	Handgrip	11.0 (45.5%)	23.0 ± 3.3	NR	Physically active	71.0 ± 10.0	23.0 ± 2.7	Normotensive
			12.0 (41.7%)	60.0 ± 6.9			81.0 ± 13.9	26.2 ± 2.4	
Greaney *et al*. (2015) [55]	United States	Handgrip	23.0 (NR)	60.0 ± 4.8	NR	NR	NR	26.7 ± 3.8	Normotensive
			15.0 (NR)	63.0 ± 3.9				27.6 ± 2.7	Hypertensive
Grossman *et al*. (1989) [56]	United States	Handgrip	18.0 (33.0%)	53.0 ± 12.0	NR	NR	NR	NR	Hypertensive
Hallman *et al*. (2011) [57]	Sweden	Handgrip	21.0 (90.5%)	40.8 ± 7.0	NR	NR	NR	24.3 ± 3.7	Normotensive
Heffernan *et al*. (2005) [58]	United States	Handgrip	10.0 (50.0%)	27.5 ± 8.5	NR	Sedentary/moderately active	75.3 ± 14.6	26.6 ± 3.5	Normotensive
Heng *et al*. (1988) [59]	United States	Handgrip	12.0 (0.0%)	29.0 ± 5.0	NR	NR	67.0 ± 5.0	NR	Normotensive
Hickey *et al*. (1993) [60]	United States	Two-knee extension	8.0 (0.0%)	24.0 ± 0.5	NR	Trained	77.6 ± 3.4	NR	Normotensive
Hirasawa *et al*. (2016) [61]	Japan	One-knee extension	12.0 (67.0%)	21.0 ± 2.0	NR	NR	58.0 ± 8.0	NR	Normotensive
Huikuri *et al*. (1986) [62]	Finland	Handgrip	13.0 (54.0%)	25.0 ± 6.0	NR	NR	NR	NR	Normotensive
Ichinose *et al*. (2006) [63]	Japan	Handgrip	13.0 (23.0%)	23.0 ± 3.6	NR	NR	62.4 ± 11.2	NR	Normotensive
Iellamo *et al*. (1993) [64]	Italy	Handgrip	10.0 (0.0%)	NR	NR	NR	NR	NR	Normotensive
Iellamo *et al*. (1999) [65]	Italy	One-knee extension	11.0 (0.0%)	26.0 ± 2.4	NR	Untrained	NR	NR	Normotensive
Incognito *et al*. (2018) [66]	Canada	Handgrip	29.0 (0.0%)	24.0 ± 5.0	NR	NR	NR	24.0 ± 3.0	Normotensive
Kadetoff and Kosek (2007) [67]	Sweden	One-knee extension	17.0 (100.0%)	37.4 ± NR	NR	NR	NR	NR	Normotensive
Kadetoff and Kosek (2010) [68]	Sweden	Two-knee extension	16.0 (100.0%)	38.3 ± NR	NR	NR	NR	NR	Normotensive
Kagaya and Homma (1997) [69]	Japan	Handgrip	7.0 (100.0%)	22.3 ± 2.9	NR	Physically active	54.4 ± 7.5	NR	Normotensive
Kahn *et al*. (1997) [70]	France	Handgrip	12.0 (0.0%)	23.6 ± 1.4	NR	NR	73.0 ± 9.4	NR	Normotensive
Kalfon *et al*. (2015) [71]	United States	Handgrip	16.0 (0.0%)	23.7 ± 6.8	NR	Sedentary	86.8 ± 14.8	29.3 ± 4.4	Normotensive
Kamiya *et al*. (2001) [72]	Japan	Handgrip	22.0 (0.0%)	22.0 ± 9.4	NR	NR	65.0 ± 9.4	NR	Normotensive
Koletsos *et al*. (2019) [73]	Greece	Handgrip	28.0 (42.9%)	43.8 ± 13.0	NR	Minimally and moderately active	NR	26.6 ± 4.1	Normotensive
			27.0 (40.7%)	47.5 ± 11.6				27.6 ± 4.7	Hypertensive (masked)
			31.0 (48.4%)	47.6 ± 7.0				26.8 ± 3.9	Hypertensive (true)
Kordi *et al*. (2012) [74]	Iran	Handgrip	20.0 (60.0%)	19.3 ± 2.0	NR	NR	NR	NR	NR
Koutnik *et al*. (2014) [75]	United States	Handgrip	20.0 (0.0%)	22.1 ± 9.0	NR	Not regularly active	84.7 ± 14.0	27.1 ± 4.5	Normotensive
Kramer *et al*. (1983) [76]	Germany	Handgrip (unilateral e (bilateral))	4.0 (0.0%)	NR	NR	NR	NR	NR	NR
Lewis *et al*. (1985) [77]	United States	Handgrip	6.0 (0.0%)	27.0 ± 3.0	NR	NR	74.60 ± 8.7	NR	Normotensive
		Two-knee extension							
Lindquist *et al*. (1973) [78]	United States	Handgrip	21.0 (0.0%)	32.0 ± NR	NR	NR	NR	NR	Normotensive
Lykidis *et al*. (2008) [79]	England	Handgrip	9.0 (44.4%)	21.8 ± 6.7	NR	Physically active	NR	NR	NR
Maiorano *et al*. (1989) [80]	Italy	Handgrip	50.0 (0.0%)	19.3 ± 1.2	NR	Trained and	68.88 ± 11.0	22.92 ± 3.2	Normotensive
			50.0 (0.0%)	19.2 ± 1.2		untrained	68.66 ± 10.2	22.99 ± 3.8	
Majahalme *et al*. (1997) [81]	Finland	Handgrip	28.0 (0.0%)	39.5 ± 4.2	NR	NR	81.7 ± 8.7	25.4 ± 2.6	Normotensive
			14.0 (0.0%)	40.7 ± 4.3			87.6 ± 10.6	26.9 ± 3.5	Hypertensive (borderline)
			24.0 (0.0%)	40.0 ± 3.9			81.9 ± 8.6	26.5 ± 2.6	Hypertensive (mild)
Mäkinen *et al*. (2008) [82]	Finland	Handgrip	10.0 (0.0%)	22.5 ± 1.6	NR	NR	72.4 ± 7.3	22.3 ± 1.6	Normotensive
Matthews *et al*. (2017) [83]	United States	Handgrip	16.0 (100.0%)	22.0 ± 3.0	NR	-	NR	22.0 ± 3.0	Normotensive
			16.0 (100.0%)	22.0 ± 2.0		-		22.0 ± 3.0	
McCoy *et al*. (1991) [84]	United States	Handgrip	9.0 (0.0%)	NR	NR	NR	71.5 ± 6.6	NR	NR
McDermott *et al*. (1974) [85]	United States	Handgrip	10.0 (0.0%)	25.3 ± 4.1	NR	Untrained	78.4 ± 7.6	NR	Normotensive
			12.0 (0.0%)	46.8 ± 2.8			80.9 ±12.5		
Metelitsina *et al*. (2010) [86]	United States	Handgrip	19.0 (63.2%)	64.7 ± 8.3	White - 18 (94.7%)	NR	NR	NR	Normotensive/Hypertensive
Mizushige *et al*. (1997) [87]	Japan	Handgrip	14.0 (42.9%)	59.0 ± NR	NR	NR	NR	NR	Normotensive
Momen *et al*. (2010) [88]	United States	Handgrip	11.0 (0.0%)	NR	NR	NR	NR	23.0 ± 1.0	Normotensive
			11.0 (100.0%)					22.0 ± 1.0	
Mortensen *et al*. (2016) [89]	England	Elbow flexion (unilateral)	75.0 (49.3%)	38.8 ± 10.9	NR	NR	NR	25.1 ± 4.4	Normotensive
Muller *et al*. (2011) [90]	United States	Handgrip	10.0 (50.0%)	25.0 ± 3.2	NR	NR	73.0 ± 12.7	NR	Normotensive
Nagle *et al*. (1988) [91]	United States	Handgrip	10.0 (0.0%)	24.0 ± 3.0	NR	Untrained	71.0 ± 10.0	NR	Normotensive
		Two-knee extension							
		Deadlift							
Nakamura *et al*. (2005) [92]	Japan	Elbow flexion (unilateral)	8.0 (0.0%)	63.0 ± 3.7	NR	NR	NR	23.1 ± 1.4	Normotensive/Hypertensive
Notay *et al*. (2018) [93]	Canada	Handgrip	200.0 (54.5%)	22.0 ± 3.0	Caucasian (non-Hispanic) = 192	Recreationally active	69.0 ± 13.0	23.0 ± 3.0	Normotensive
					Hispanic = 5				
					Black = 3				
Notay *et al*. (2018b) [94]	Canada	Handgrip	66.0 (0.0%)	22.0 ± 3.0	NR	Recreationally active	77.0 ± 13.0	24.0 ± 3.0	Normotensive
			66.0 (100.0%)	21.0 ± 2.0			63.0 ± 9.0	23.0 ± 3.0	
Nyberg (1976) [95]	Australia	Handgrip	10.0 (0.0%)	30.6 ± NR	NR	NR	NR	NR	Normotensive
			9.0 (100.0%)	30.4 ± NR					Hypertensive (untreated)
			9.0 (0.0%)	45.3 ± NR					Hypertensive (treated)
			12.0 (100.0%)	46.8 ± NR					
			12.0 (0.0%)	46.9 ± NR					
			5.0 (100.0%)	48.4 ± NR					
Park *et al*. (2012) [96]	United States	Handgrip	12.0 (33.3%)	28.9 ± 4.9	Caucasia= 6	NR	62.8 ± 8.0	21.7 ± 1.7	Normotensive
			12.0 (41.7%)	32.3 ± 7.6	Hispanic= 3		82.9 ± 11.1	27.4 ± 1.4	
					Asian= 3				
					Caucasian= 7				
					Hispanic = 4				
					Asian= 1				
Parmar *et al*. (2018) [23]	Canada	Handgrip	11.0 (0.0%)	24.0 ± 3.3	NR	Physically active	75.0 ± 6.6	23.7 ± 1.7	Normotensive
			9.0 (100.0%)	22.0 ± 3.0			61.0 ± 3.0	22.0 ± 1.5	
			10.0 (100.0%)	22.0 ± 6.3			61.0 ±12.7	22.3 ± 4.1	
Pepin *et al*. (1996) [97]	United States	Handgrip	25.0 (64.0%)	34.3 ± 5.5	NR	NR	NR	NR	NR
Petrosfsky and Laymon (2002) [98]	United States	Handgrip	20–30 years = 15.0 (NR)	NR	NR	Untrained	81.8 ± NR	NR	NR
		Two-knee extension	31–40 years = 10.0 (NR)				83.4 ± NR		
			41–50 years = 12.0 (NR)				83.5 ± NR		
			51–65 years = 13.0 (NR)				85.1 ± NR		
Piccolino *et al*. (2018) [99]	Italy	Handgrip	25.0 (8.0%)	43.2 ± 8.3	Caucasian	NR	NR	NR	Normotensive
Plotnikov *et al*. (2002) [100]	Russia	Handgrip	48.0 (100.0%)	NR	NR	NR	NR	NR	Normotensive
		Torso effort							
Quarry and Spodick (1974) [101]	United States	Handgrip	10.0 (0.0%)	NR	NR	Physically active	NR	NR	Normotensive
Riendl *et al*. (1977) [102]	United States	Finger adduction	10.0 (0.0%)	25.1 ± 2.2	NR	Untrained	NR	NR	Normotensive
		Plantar flexion							
Sagiv *et al*. (1985) [103]	United States	Handgrip	10.0 (0.0%)	52.0 ± 2.0	NR	NR	NR	NR	Normotensive
		Deadlift							
Sagiv *et al*. (1988) [104]	Israel	Deadlift	10.0 (0.0%)	28.0 ± 3.0	NR	Physically active	82.0 ± 3.0	NR	Normotensive
			10.0 (0.0%)	67.0 ± 4.0			80.0 ± 2.0		
Sagiv *et al*. (1988b) [105]	Israel	Deadlift	25.0 (0.0%)	27.4 ± 2.3	NR	Physically active	82.3 ± 10.9	NR	Normotensive
			25.0 (0.0%)	51.0 ± 3.2			79.5 ± 7.6		
			25.0 (0.0%)	67.8 ± 3.8			80.0 ± 10.2		
Sagiv *et al*. (1988c) [106]	Israel	Handgrip	10.0 (0.0%)	28.0 ± 3.0	NR	Physically active	81.7 ± 3.1	NR	Normotensive
		Deadlift	10.0 (0.0%)	67.0 ± 4.0			79.5 ± 2.4		
Sagiv *et al*. (1995) [107]	United States	Handgrip	5.0 (0.0%)	33.0 ± 5.0	NR	Physically active	NR	NR	Normotensive
		Deadlift							
Sagiv *et al*. (2008) [108]	Israel	Deadlift	15.0 (0.0%)	40.0 ± 13.0	NR	NR	80.5 ± 9.2	NR	Normotensive
Samora *et al*. (2019) [109]	Brazil	Handgrip	20.0 (0.0%)	21.0 ± 2.7	NR	Physically active	78.0 ± 9.8	24.9 ± 2.7	Normotensive
			20.0 (100.0%)	23.0 ± 2.7			61.4 ± 9.8	23.0 ± 2.7	
Seals (1989) [110]	United States	Handgrip (unilateral and bilateral)	9.0 (33.0%)	NR	NR	NR	NR	NR	Normotensive
Seals *et al*. (1983) [111]	United States	Elbow extension	6.0 (0.0%)	NR	NR	Untrained and trained (untrained and trained members after a training period)	Untrained	NR	Normotensive
		One-knee extension					72.7 ± 13.1 Trained		
							71.7 ± 13.9		
Seals *et al*. (1985) [112]	United States	Handgrip	10.0 (40.0%)	62.0 ± 1.0	NR	Untrained and trained	Before: 74.0 ± 12.0 After: 73.0 ± 11.0	NR	Normotensive
Somani *et al*. (2018) [22]	Canada and England	Handgrip	26.0 (50.0%)	25.0 ± 4.0	NR	Recreationally active/non-active	72.0 ± 15.0	24.0 ± 4.0	Prehypertensive/Normotensive
		Two-knee extension	20.0 (50.0%)	22.0 ± 4.0	NR		73.0 ± 14.0	25.0 ± 4.0	
Stewart *et al*. (2007) [113]	United States	Handgrip	16.0 (56.3%)	24.5 ± NR	NR	NR	70.0 ± 14.0	24.0 ± 4.0	Normotensive
Tan *et al*. (2013) [114]	United States	Handgrip	11.0 (45.5%)	25.0 ± 3.0	NR	NR	NR	NR	Normotensive
Taylor *et al*. (2017) [115]	England	Wall squat	25.0 (0.0%)	44.6 ± 1.7	NR	Physically inactive	89.1 ± 2.4	NR	Prehypertensive
Turley (2005) [116]	United States	Handgrip	35.0 (0.0%)	20.2 ± 2.1	NR	Untrained	78.1 ± 10.1	24.6 ± 2.9	Normotensive
			35.0 (100.0%)	19.9 ± 1.8			62.8 ± 8.5	23.0 ± 2.6	
Umeda *et al*. (2009) [117]	United States	Handgrip	23.0 (100.0%)	20.0 ± 2.0	NR	Physically active	NR	NR	Normotensive
Umeda *et al*. (2015) [118]	United States	Handgrip	14.0 (36.0%)	22.1 ± 2.9	African-Americans	Recreationally active	NR	26.02 ± 3.1	Normotensive
			14.0 (36.0%)	21.9 ± 3.0	White (non-Hispanic)			24.06 ± 3.4	
Van Huysduynen *et al*. (2004) [119]	Netherlands	Handgrip	41.0 (0.0%)	32.6 ± 11.2	NR	Untrained/Trained	NR	NR	Normotensive
Vaz *et al*. (1993) [120]	India	Handgrip	8.0 (NR)	NR	NR	NR	NR	NR	Normotensive
Vianna *et al*. (2012) [121]	Brazil	Handgrip	8.0 (0.0%)	25.0 ± 2.0	NR	NR	78.0 ± 11.0	NR	Normotensive
Vitcenda *et al*. (1990) [122]	United States	Deadlift	16.0 (0.0%)	27.0 ± 6.0	NR	Untrained	75.0 ± 8.0	NR	NR
Weippert *et al*. (2013) [123]	Germany	Leg press	23.0 (0.0%)	25.5 ± 2.6	NR	Physically active	84.0 ± 7.7	24.3 ± 1.5	Normotensive
Wiles *et al*. (2018) [13]	England	Wall squat	26.0 (0.0%)	45.0 ± 8.0	NR	Physically inactive	89.7 ± 12.3	NR	Hypertensive
Williams (1991) [124]	United States	Handgrip	6.0 (0.0%)	26.0 ± 3.0	NR	NR	NR	NR	NR
		Two-knee extension							
Wright *et al*. (1999) [125]	United States	One-knee extension	15.0 (0.0%)	21.6 ± 1.2	African-American	NR	82.5 ± 19.8	NR	Normotensive
			15.0 (100.0%)	27.7 ± 6.2	Asian American		62.1 ± 7.4		
			15.0 (0.0%)	27.8 ± 7.4	Caucasian American		69.0 ± 7.4		
			15.0 (100.0%)	27.0 ± 6.2			54.7 ± 5.4		
			15.0 (0.0%)	26.4 ± 7.0			83.2 ± 8.5		
			15 (100%)	25.2 ± 6.6			60.0 ± 10.5		
Yamaji *et al*. (1983) [19]	Japan	Elbow flexion	20.0 (0.0%)	20.4 ± 1.5	NR	NR/Trained	64.8 ± 8.2	NR	Normotensive
		One-knee extension							

Note: Data presented as mean ± standard deviation. BMI, body mass index; NR, not reported.

The total number of participants was 2695, with a mean age ranging from 19.2 to 73.0 years. Most of the studies included only men (47.1%). More than half of the studies (56.9%) did not report the trainability status of the participants, and among the studies that reported this information, only 18.2% included trained participants and/or athletes.

Regarding BP level classification, 76.5% included only normotensive participants. In addition, only eight studies reported information regarding the number of users of antihypertensive medications. Regarding BP measurement protocols during exercise, the auscultatory, automatic, and finger photoplethysmography (Finometer) methods presented similar frequencies in the studies (30%). Concerning the moment of BP measurement, 66 studies (64.7%) performed it at the end of the exercise contraction, with 21 studies reporting that this measurement was performed in the final minute or final seconds of exercise, but it is not clear at what exact time this was done. In the other studies, the BP measurement was taken at different moments during exercise.

### 3.3 Characteristics of Exercise Protocols

Most studies used a single set (72.6%) and performed sets lasting up to 180 seconds (74%). Regarding exercise intensity, 61.9% of the studies performed sets with low intensities (i.e., ≤30% MVC) (**Supplementary Material 2**).

### 3.4 Overall Effect of Different Types of Isometric Exercise on Blood Pressure Response

All the details regarding the BP responses to the handgrip or other IE are shown in the **Supplementary Material 3, 4, 5 and 6**.

Table 2 shows the overall effects for each type of IE on the BP response. The greater increases in SBP were +64.5 mmHg (*p *≤ 0.001) for the two-knee extension, +61.6 mmHg (*p *≤ 0.001) for the deadlift, and +51.5 mmHg (*p *≤ 0.001) for the leg press. These increases were higher than those for one-knee extension, plantar flexion, and torso effort exercises. The mean increases identified for the two-knee extension and deadlift exercises were statistically greater than those identified for the handgrip. For DBP, the greater increases were +52.2 mmHg (*p *≤ 0.001) for the two-knee extension, and +43.4 mmHg; (*p *≤ 0.001) for the squat. Differences were identified when the handgrip is compared to the two-knee extension, squat, and deadlift exercises. Moreover, statistical differences were also observed between the two-knee extension and deadlift exercises.

**Table 2.** **Overall effects of different types of isometric exercise on blood pressure response**.

Type of exercise	N	Mean difference	Standard error	Variance	95% CI	Z-value	p*	$I^{2}$	* $p^{¥}$ *
SBP (mmHg)									
Handgrip	127	+33.4	1.8	3.2	29.9–36.9	18.6	0.0	99.2	0.0
Elbow flexion	8	+47.3	12.8	163.7	22.2–72.4	3.7	0.0	99.1	0.0
One-knee extension	17	+34.3	2.1	4.3	30.2–38.3	16.4	0.0	84.7	0.0
Two-knee extension	11	+64.5	5.9	35.2	52.8–76.1	10.9	0.0	96.1	0.0
Leg press	4	+51.5	11.0	121.1	29.9–73.0	4.7	0.0	94.7	0.0
Squat	3	+46.3	10.9	117.8	25.0–67.5	4.3	0.0	97.1	0.0
Plantar flexion	2	+23.3	4.0	15.9	15.5–31.1	5.8	0.0	53.4	0.1
Deadlift	13	+61.6	2.7	7.2	56.4–66.9	22.9	0.0	66.4	0.0
Torso effort	3	+20.8	6.9	47.8	7.2–34.3	3.0	0.0	99.9	0.0
DBP (mmHg)									
Handgrip	112	+25.1	1.0	1.1	23.0–27.1	24.0	0.0	98.4	0.0
Elbow flexion	8	+22.4	2.7	7.6	17.0–27.7	8.1	0.0	83.8	0.0
One-knee extension	17	+26.4	1.9	3.6	22.7–30.1	14.0	0.0	87.3	0.0
Two-knee extension	11	+52.2	5.4	29.5	41.5–62.8	9.6	0.0	97.3	0.0
Leg press	4	+34.4	8.1	66.1	18.4–50.3	4.2	0.0	92.2	0.0
Squat	2	+43.4	6.5	42.2	30.7–56.2	6.7	0.0	94.5	0.0
Plantar flexion	2	+22.4	1.9	3.6	18.7–26.2	11.8	0.0	0.0	0.4
Deadlift	13	+34.4	1.9	3.7	30.6–38.1	17.8	0.0	79.0	0.0
Torso effort	3	+23.8	3.2	10.4	17.5–30.1	7.4	0.0	99.6	0.0

Note: Analyses performed with the random effects model. N, number of studies and subgroups per study analyzed; CI, confidence interval; $I^{2}$, heterogeneity of studies. For the plantar flexion and torso effort exercises only one study was included in the analysis. *p concerns the main analysis (mean difference). ^¥^*p* concerns the heterogeneity analysis ($I^{2}$).

For SBP, the largest differences were found between two-knee extension and torso effort (–48.6 mmHg; *p *< 0.001), two-knee extension and plantar flexion (–46.4 mmHg; *p *< 0.001). For the handgrip, the greatest differences were against two-knee extension (+36.1 mmHg; *p *< 0.001) and deadlift (+26.6 mmHg; *p *< 0.001). Regarding DBP, the largest differences were observed between two-knee extension and plantar flexion (–34.2 mmHg; *p *< 0.001), elbow flexion and two-knee extension (+33.0 mmHg; *p *< 0.001). For the handgrip, the greatest differences were against two-knee extension (+31.4 mmHg; *p *< 0.001) (**Supplementary Material 7**).

### 3.5 Effect of Comparing Handgrip and Two-Knee Extension Exercises

**Fig. 2.**
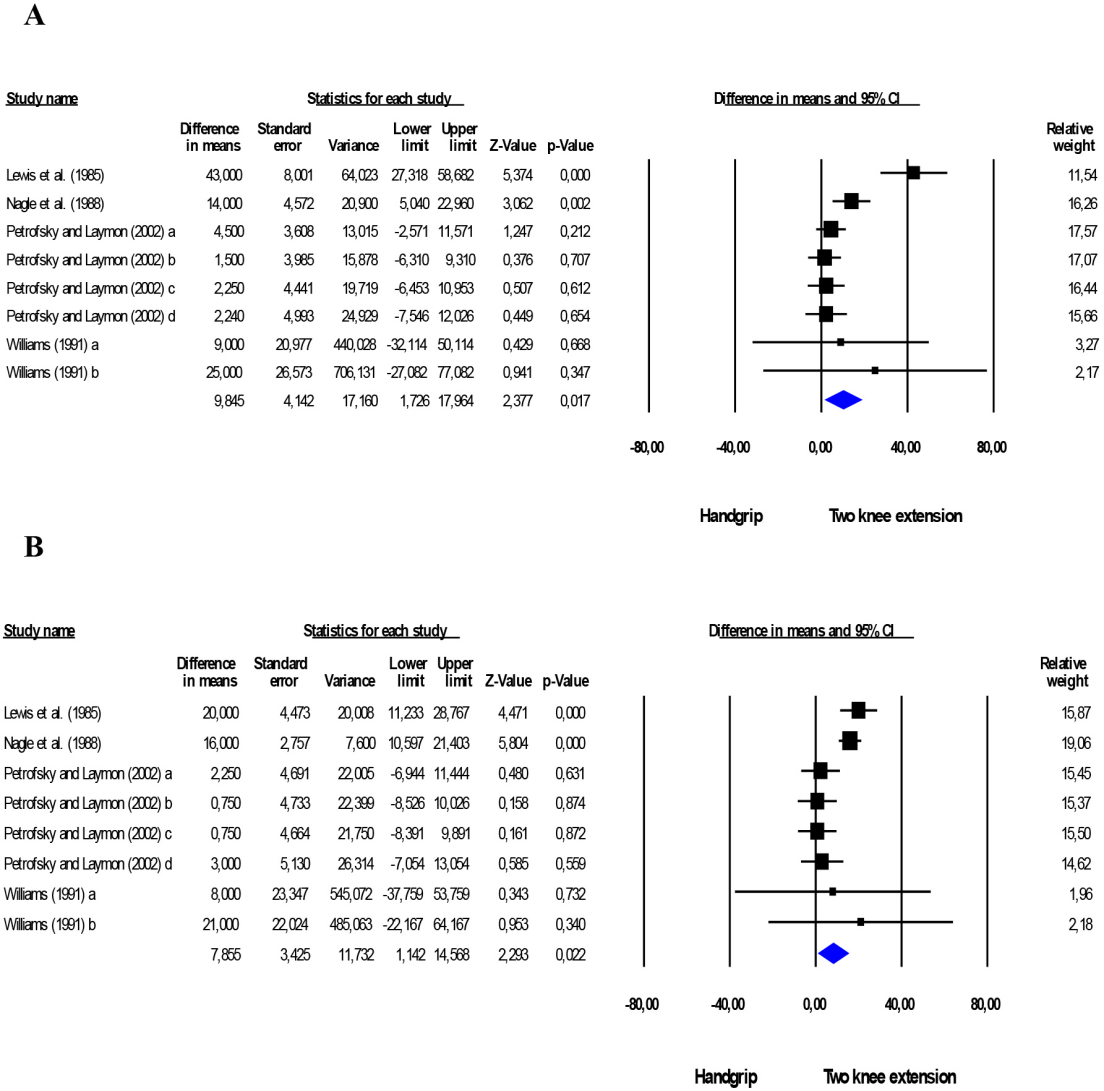
**Comparison between isometric handgrip and two-knee extension exercises**. Mean difference in systolic (A) and diastolic (B) BP between isometric handgrip and two-knee extension exercises. Estimation per study (black square). Overall estimate from random effects analyses (blue diamond). 95% CI indicates confidence interval. $I^{2}$ indicates the heterogeneity of the studies.

Two-knee extension promoted greater increases in SBP (+9.8 mmHg; *p* = 0.017; $I^{2}$ = 74.5%, *p *≤ 0.001) and DBP (+7.9 mmHg; *p* = 0.022; $I^{2}$ = 68.6%, *p* = 0.002) compared to handgrip (Fig. 2). When performing sensitivity analysis, removing the study by Lewis *et al*. [77] from the meta-analysis, there was a reduction of the effect for SBP (+4.9 mmHg; *p* = 0.01; $I^{2}$ = 0%, *p* = 0.429) and DBP (+7.9 mmHg; *p *≤ 0.001; $I^{2}$ = 62.5%, *p* = 0.014).

### 3.6 Effect of Comparing Handgrip and Deadlift Exercises

**Fig. 3.**
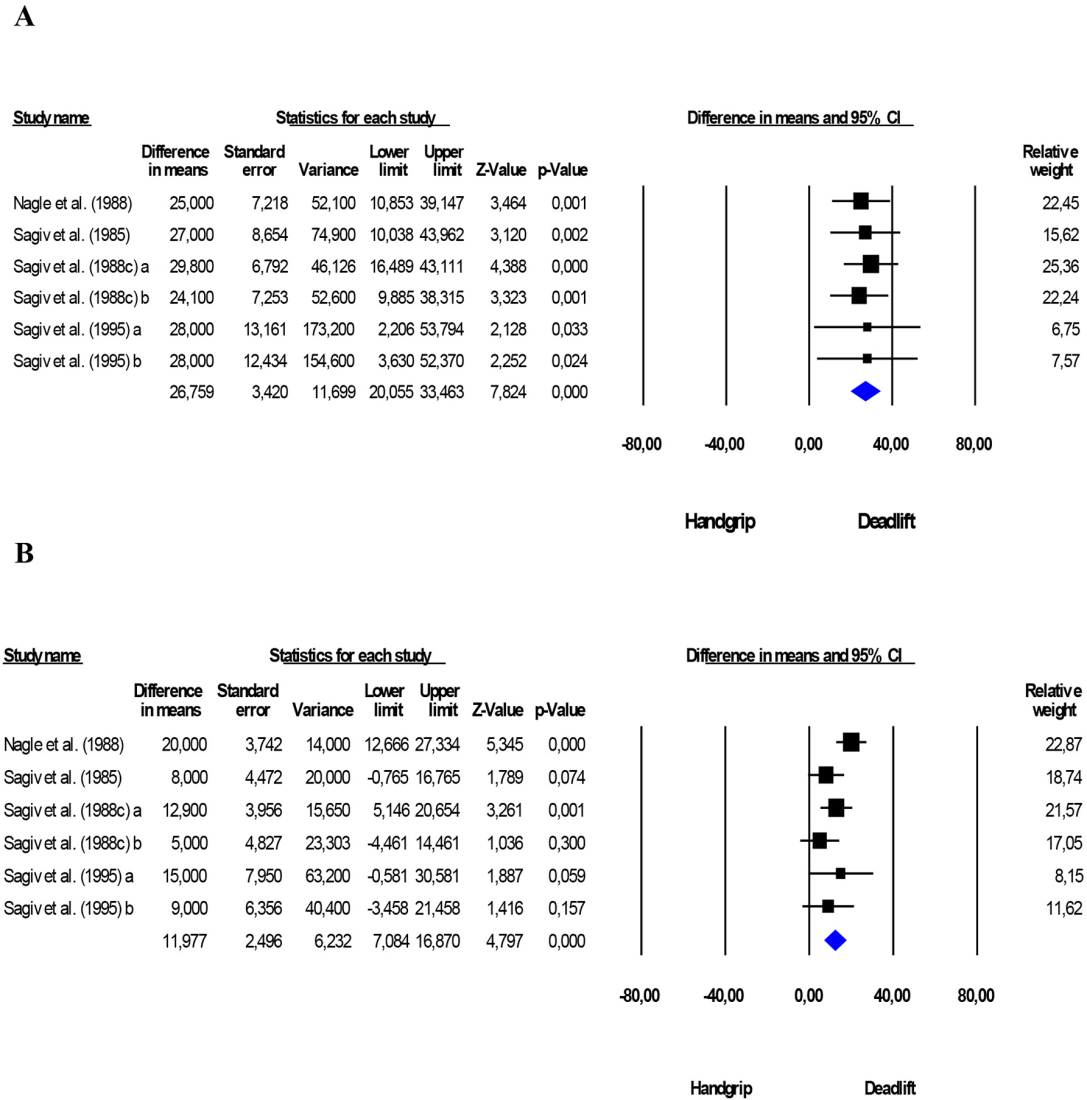
**Comparison between isometric handgrip and deadlift exercises**. Mean difference in systolic (A) and diastolic (B) BP between isometric handgrip and land lift exercises. Estimation per study (black square). Overall estimate from fixed effects analyses (blue diamond). 95% CI indicates confidence interval. $I^{2}$ indicates the heterogeneity of the studies.

Comparing handgrip and deadlift, greater increases were observed in SBP (+26.8 mmHg; *p *≤ 0.001; $I^{2}$ = 0%, *p* = 0.995) and DBP (+12.4 mmHg; *p *≤ 0.001; $I^{2}$ = 36.3%, *p* = 0.165) for the deadlift (Fig. 3).

### 3.7 Effect of Handgrip Exercise on Blood Pressure Response according to Participants Characteristics

For SBP, men (+34.5 mmHg; *p *≤ 0.001), middle-aged/elderly adults (+41.3 mmHg; *p *≤ 0.001), and hypertensive individuals (+39.6 mmHg; *p *≤ 0.001) showed greater increases than their peers. For DBP, men (+26.6 mmHg; *p *≤ 0.001) and middle-aged/elderly adults (+29.6 mmHg; *p *≤ 0.001) presented higher increases than their peers. Analyzing only the studies that directly compared men and women for handgrip [23, 95, 109] it was observed greater increases for men only in DBP (+4.2 mmHg; *p* = 0.017, $I^{2}$ = 9.5%, *p* = 0.356) (Table 3).

**Table 3.** **Effect of isometric handgrip exercise on blood pressure response according to participants’ characteristics**.

Subgroup		N	Mean difference	Standard error	Variance	95% CI	Z-value	p*	$I^{2}$	* $p^{¥}$ *
SBP (mmHg)										
Sex										
	Men	59	+34.5	2.1	4.5	30.3–38.6	16.2	0.0	94.6	0.0
	Women	14	+26.1	3.9	15.2	18.4–33.7	6.7	0.0	99.6	0.0
Age										
	Young	62	+31.3	2.1	4.5	27.2–35.5	14.7	0.0	95.9	0.0
	Middle-aged/elderly	37	+41.3	2.1	4.4	37.1–45.4	19.6	0.0	95.0	0.0
BP level classification										
	Non-hypertensive	95	+30.7	2.1	4.3	26.7–34.8	14.9	0.0	99.3	0.0
	Hypertensive	13	+39.6	2.2	4.7	35.3–43.8	18.2	0.0	71.8	0.0
DBP (mmHg)										
Sex										
	Men	50	+26.6	3.1	9.5	20.5–32.6	8.6	0.0	98.4	0.0
	Women	14	+20.4	2.9	8.4	14.7–26.0	7.0	0.0	99.3	0.0
Age										
	Young	55	+23.4	1.5	2.3	20.4–26.3	15.4	0.0	94.7	0.0
	Middle-aged/elderly	36	+29.6	2.6	6.6	24.6–34.6	11.5	0.0	98.8	0.0
BP level classification										
	Non-hypertensive	80	+22.1	1.0	1.0	20.2–24.1	22.6	0.0	97.9	0.0
	Hypertensive	13	+30.8	8.9	78.4	13.5–48.2	3.5	0.0	99.5	0.0

Note: Analyses performed with the random effects model. N, number of studies and subgroups per study analyzed; Young, studies that included adults with mean age up to 40 years; Middle-aged/elderly, studies that included adults with a mean 40 years; Non-Hypertension, studies that classified participants into normotensives and/or prehypertensive; CI, confidence interval; $I^{2}$, heterogeneity of studies. *p concerns the main analysis (mean difference).^¥^ *p* concerns the heterogeneity analysis $I^{2}$).

### 3.8 Effect of Handgrip Exercise on Blood Pressure Response according to the Characteristics of Exercise Protocols

Higher intensities (>60% MVC) demonstrated the largest absolute increases in SBP (+55.8 mmHg; *p *≤ 0.001) and DBP (+52.4 mmHg; *p *≤ 0.001) compared to lower intensities (≥30% MVC) and similar increases compared to >30 and ≤60% of MVC. Intensities between >30 and ≤60% promoted greater increases for SBP (+40.7 mmHg; *p *≤ 0.001) and DBP (+31.9 mmHg; *p *≤ 0.001) compared to lower intensities. Acute BP responses to IE were similar when compared the different contraction durations (≤ 120 > 120 e ≤ 180 e >180 seconds) (Table 4).

**Table 4.** **Effect of isometric handgrip exercise on blood pressure response according to the characteristics of the exercise protocols**.

Subgroup		N	Mean difference	Standard error	Variance	95% CI	Z-value	p*	$I^{2}$	* $p^{¥}$ *
SBP (mmHg)										
Intensity										
	≤ 30%	76	+27.5	1.7	2.9	24.2–30.9	16.3	0.0	98.6	0.0
	> 30 e ≤ 60%	44	+40.7	1.9	3.5	37.0–44.3	21.8	0.0	92.7	0.0
	>60%	7	+55.8	9.1	83.3	37.9–73.7	6.1	0.0	92.9	0.0
Duration										
	≤120	45	+35.5	2.6	6.8	30.4–40.7	13.6	0.0	96.6	0.0
	> 120 e ≤ 180	48	+32.6	2.0	3.9	28.7–36.5	16.5	0.0	94.5	0.0
	>180	27	+33.6	3.1	9.3	27.6–39.6	11.0	0.0	99.4	0.0
DBP (mmHg)										
Intensity										
	≤30%	69	+20.1	1.6	2.5	17.0–23.2	12.6	0.0	98.7	0.0
	> 30 e ≤ 60%	39	+31.9	1.5	2.2	29.0–34.8	21.4	0.0	93.8	0.0
	>60%	4	+52.4	11.9	141.0	29.1–75.6	4.4	0.0	94.1	0.0
Duration										
	≤120	42	+24.5	1.4	1.9	21.8–27.2	17.9	0.0	94.2	0.0
	> 120 e ≤ 180	42	+26.8	3.1	9.6	20.8–32.9	8.6	0.0	98.6	0.0
	>180	21	+24.5	2.5	6.1	19.6–29.3	9.9	0.0	99.1	0.0

Note: Analyses performed with the random effects model. N, number of studies and subgroups per study analyzed; Intensity, percentage of MVC or MR; Duration, contraction time in seconds; CI, confidence interval; $I^{2}$, heterogeneity of studies. *p concerns the main analysis (mean difference).^¥^ *p* concerns the heterogeneity analysis ($I^{2}$).

### 3.9 Risk of bias

**Fig. 4.**
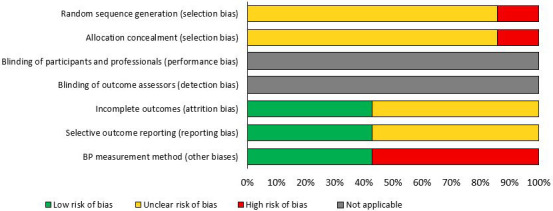
**Risk of bias analysis of studies that compared the BP response to handgrip exercise and other types of isometric exercise (n = 7)**.

Fig. 4 describes the risk of bias for the seven studies included in the meta-analyses comparing BP response to handgrip and other IE.

## 4. Discussion

This study showed that exercises involving large muscle groups promoted the highest increases in BP among all IE types. These findings support the hypothesis that muscle mass interferes with the BP response to IE [27, 110, 126] possibly because of the greater activation of the central command, intramuscular pressure, and vascular occlusion generated [111, 127]. However, this relationship is still controversial since some studies suggest that the size of the muscle is not a determining factor for BP responses [42, 124], which are mainly influenced by the magnitude of the force exerted during contraction, especially when high percentages are reached [128].

Although the overall results of the present study for each IE alone showed higher increases for the exercises involving larger muscle groups, important characteristics of the exercise protocols, such as intensity, were not considered in the analyses. Thus, some studies adopting higher intensities may have accentuated these overall BP responses, since few studies were included in the analyses and the heterogeneity among them was high. Otherwise, in the analyses comparing handgrip and two-knee extension and deadlift exercises, the exercise protocols used were similar, which reduces the possible effect of the intensity and reinforces the role of muscle mass on the BP response.

Although the exercises with larger muscle groups showed greater increases than those with smaller muscle masses, when analyzing the studies individually, only the study by Williams [124] promoted an average increase in SBP above 250 mmHg, which is the cutoff point considered safe. However, this study performed an intensity of 100% MVC, had a small sample size and measured BP with the intra-arterial method, which affect the BP response identified. Moreover, adopting 120 mmHg as the safety value for DBP [129], some studies that showed values higher than this limit included hypertensive participants [31, 81, 95], high intensity exercise [44, 101, 124], long duration of contraction (above 120 seconds) [60, 98], very small sample sizes (6 and 7 participants), and sedentary individuals performing six sets of the exercise [42]

In the subgroup analyses, men showed higher increases for SBP and DBP in response to handgrip than women. It could be explained by the fact that the majority of studies included young men and women, since premenopausal women seem to present attenuation of sympathetic nervous activity, catecholamine release, mechanoreflex, and the degree of vasoconstriction during exercise compared to men of the same age [130, 131]. Otherwise, analyzing the studies that directly compared men and women, greater increases were observed for men only for DBP.

Furthermore, middle-aged/elderly adults showed higher mean increases for SBP and DBP than younger adults for the handgrip exercise. The elevated pressure response with age is still not a consensus, since some studies suggest that there is no exacerbation of this mechanism during healthy aging. However, it is known that the aging process is associated with several structural, hormonal, and functional changes, including increased arterial stiffness, peripheral vascular resistance, and sympathetic activity, as well as deterioration of endothelial function [132], which increases the risk of developing hypertension with advancing age [133]. Thus, in studies that included older participants, the prevalence of hypertension was also higher, which would help to explain, in part, these findings.

Higher increase in SBP was observed for hypertensive compared to non-hypertensive individuals during handgrip exercise, but not for DBP. Such response was expected since hypertensive individuals present autonomic imbalance, with sympathetic hyperactivation [134]. Nevertheless, it must be emphasized that we included in this review studies with medicated and non-medicated hypertensive individuals. The use of different classes of antihypertensive medications, at different times of the day, may have influenced the BP responses to IE. However, it was not possible to perform an analysis considering this variable due to the lack of information available in the studies.

Regarding the characteristics of the exercise protocol, only intensity influenced SBP and DBP during handgrip. These findings support the hypothesis that higher intensities promote BP responses to exercise [20, 128]. Although the studies with high intensities (>60% MVC) showed higher increases for SBP and DBP than those with moderate intensities (>30 and ≤60% MVC), these were not significantly different. However, it is believed that this result is explained, in part, by the small number of studies included in the analyses with high intensities and also by the high heterogeneity among them.

Concerning the practical application of the present study, it should be considered that even those IE that involve greater muscle mass do not seem to bring great cardiovascular risks to the practitioner. Such findings contradict our initial hypothesis that exercise involving large muscle groups would cause exaggerated responses in BP. On the other hand, those exercises with smaller muscle masses promoted lower BP responses, proving to be even safer from the cardiovascular point of view. Furthermore, during handgrip exercise, it is relevant to have a special attention for men, hypertensive and elderly population, and for the exercise performed at higher intensities (>60% MVC). Although subgroup analyses have not been performed for the other types of exercises, it is believed that this attention is also applicable to them, especially those involving larger muscle masses. However, further investigations are needed to confirm.

Therefore, when using IE as a strategy for the treatment of hypertension, it is necessary to considerer some characteristics of the patient. For those hypertensive individuals controlled by medication and/or who do not have other comorbidity, the choice of the type of IE is more flexible, and exercises with different muscle masses can be adopted, as long as the general precautions regarding the prescription of exercises for hypertensive individuals are taken (i.e., avoid the Valsalva maneuver during the effort). However, if the hypertensive individual is not controlled and/or presents complications or comorbidities, it seems more cautious to choose exercises involving smaller muscle masses.

Considering this, IE can be considered as a complementary non-pharmacological strategy for the prevention and treatment of hypertension in public health recommendations. However, more studies are needed to ensure the cardiovascular safety of different types of this exercise and, thus, to add it in exercise guidelines to the same extent as dynamic resistance exercise [35].

This systematic review has some limitations. The studies included in this review were conducted at different time periods and considered different guidelines for classifying subjects as hypertensive, which may result in different criteria for classifying hypertension. This, however, cannot be corrected considering BP means, since these must be influenced by antihypertensive medications. The heterogeneity among the majority of studies was high ($I^{2}$>75%), which reduces the validity of combining the individual results of the studies. Indirect comparisons were made between different exercise types. However, there is a need for direct randomized controlled trials. Moreover, few studies were included for the analysis of the comparison between handgrip exercise and other types of exercise, and it is necessary to include more studies with greater homogeneity in order to obtain more consistent results. A lack of standardization regarding when BP was acutely measured is a limitation of this work, since the studies took this measurement at different moments. Furthermore, there is a lack of exploration of study-level moderators that may influence heterogeneity, such as MVC for handgrip. The subgroup analyses were performed only for the handgrip exercise, due to the small number of studies with other exercise types. It is also important to note that the analyses were performed considering sex and age separately, therefore, it was not possible to describe the results for men and women stratified by age due to the small number of studies that included participants with these characteristics.

The strength of the present study is its originality, since this is the first systematic review with meta-analysis that sought to investigate the BP responses during the performance of different types of IE and to compare them with handgrip. Considering this, it was not possible to compare the findings of this review with those of other systematic reviews.

## 5. Conclusions

In conclusion, IE involving larger muscle groups elicit greater BP responses than those involving smaller muscle masses, especially in men, middle-aged/elderly adults and hypertensive individuals. The present study supports the literature regarding the cardiovascular safety of IE involving small muscle groups, especially at low intensities, and shed light on the investigation regarding cardiovascular safety during the performance of other types of IE in adults. However, due to the high heterogeneity of the studies, the results of this systematic review should be interpreted with caution, and further investigations are needed. Prospective studies should directly compare BP responses during various types of IE in different populations and different exercise protocol.

## Supplementary Material

Supplementary material associated with this article can be found, in the online version, at https://doi.org/10.31083/j.rcm2402060.
