# Supplementary material for: Acute Blood Pressure Response to Different Types of Isometric Exercise: A Systematic Review with Meta-Analysis
Source: Rev Cardiovasc Med. 2023 Feb 10;24(2):60. doi: 10.31083/j.rcm2402060 (PMC11273125; doi:10.31083/j.rcm2402060)
Supplement: Supplementary file 1 [file 2153-8174-24-2-060-s1.zip › Supplementary material 1.docx]

**Search Strategy**

|  | **Terms** |
| --- | --- |
| Intervention | (“Isometric Contraction” OR “Contraction, Isometric” OR “Contractions, Isometric” OR “Isometric Contractions” OR “Exercise, Isometric” OR “Exercises, Isometric” OR “Isometric Exercises” OR “Isometric Exercise” OR “Isometric Stretching” OR “Stretching, Isometric” OR "Static Contraction" OR "Contraction, Static" OR “Isometric Resistance Exercise”) |
| Outcome | (“Blood Pressure” OR “Pressure, Blood” OR “Diastolic Pressure” OR “Pressure, Diastolic” OR “Systolic Pressure” OR “Pressure, Systolic” OR “Pressures, Systolic”) |
| General | ((“Isometric Contraction” OR “Contraction, Isometric” OR “Contractions, Isometric” OR “Isometric Contractions” OR “Exercise, Isometric” OR “Exercises, Isometric” OR “Isometric Exercises” OR “Isometric Exercise” OR “Isometric Stretching” OR “Stretching, Isometric” OR "Static Contraction" OR "Contraction, Static" OR “Isometric Resistance Exercise”)) AND ((“Blood Pressure” OR “Pressure, Blood” OR “Diastolic Pressure” OR “Pressure, Diastolic” OR “Systolic Pressure” OR “Pressure, Systolic” OR “Pressures, Systolic”)) |
