# Supplementary material for: Acute Blood Pressure Response to Different Types of Isometric Exercise: A Systematic Review with Meta-Analysis
Source: Rev Cardiovasc Med. 2023 Feb 10;24(2):60. doi: 10.31083/j.rcm2402060 (PMC11273125; doi:10.31083/j.rcm2402060)
Supplement: Supplementary file 1 [file 2153-8174-24-2-060-s1.zip › Supplementary material 2.docx]

| **Characteristics of exercise protocols in different types of isometric exercise.** | | | | | |  |
| --- | --- | --- | --- | --- | --- | --- |
| **Author and year** | **Modality** | **Number of sets** | **Duration of sets (s)** | **Interval between sets (s)** | **Intensity** | |
| Almeida et al.  (2021) [10] | Handgrip | 1 | 180.0 | - | 30% MVC | |
| Aoki et al.  (1983) [40] | Handgrip | 1 | 180.0 | - | 30% MVC | |
| Bakke et al.  (2007) [42] | Handgrip | 1 | 120.0 | - | 40% MVC | |
| Bakke et al.  (2009) [43] | Handgrip | 1 | 120.0 | - | 40% MVC | |
| Balmain et al.  (2016) [41] | Handgrip | 1 | 180.0 | - | 40% MVC | |
| Bentley and Thomas (2018) [44] | Handgrip | 4 | 120.0 | 60,0 | 30% MVC | |
| Borghi et al.  (1988) [46] | Handgrip | 1 | 180,.0 | - | 30% MVC | |
| Bosisio et al.  (1980) [47] | Handgrip | 2 | 1^st^: 300.0 2^nd^: 300.0 | 1.800,0 | 1^st^: 50% MVC 2^nd^: 100% MVC | |
|  |  |  |  |  |  |  |
| Cottone et al.  (1998) [48] | Handgrip | 1 | 180.0 | - | 50% MVC | |
| Ehsani et al.  (1981) [51] | Handgrip | 3 | 1^st^: 120.0 2^nd^: 120.0 3^rd^: 45.0 | 180,0 | 1^st^: 20% MVC 2^nd^: 40% MVC 3^rd^: 60% MVC | |
| Ehsani et al.  (1982) [502] | Handgrip | 2 | 1^st^: 120.0 2^nd^: 45.0 | 180,0 | 1^st^: 40% MVC 2^nd^: 60% MVC | |
|  |  |  |  |  |  |  |
| Ferguson and Brown (1997) [53] | Handgrip | 1 | 140 ± 11.1 122 ± 14.2 | - | 40% MVC | |
| Fu et al.  (1981) [54] | Handgrip | 1 | 180.0 | - | 50% MVC | |
|  |  |  |  |  |  |  |
| Fu et al.  (2002) [55] | Handgrip | 1 | 183.0 ± 21.0 | - | 40% MVC | |
| Gois et al.  (2019) [57] | Handgrip | 1 | Supine  123.0 ± 28.0 Sitting  112.0 ± 21.0 Standing  117.0 ± 24.0 | - | 30% MVC | |
| Goldstein and Shapiro  (1988) [58] | Handgrip | 1 | 60.0 | - | 20% MVC | |
| Goldstraw and Warren  (1985) [59] | Handgrip | 3 | Max (NR) | NR | 1^st^: 10% MVC 2^nd^: 20% MVC 3^rd^: 30% MVC | |
| Goulopoulou et al (2010) [60] | Handgrip | 1 | 180.0 | - | 30% MVC | |
| Graafsma  (1989) [61] | Handgrip | 1 | 180.0 | - | 30% MVC | |
|  |  |  |  |  |  |  |
| Greaney et al.  (2013) [62] | Handgrip | 1 | 120.0 | - | 30% MVC | |
| Greaney et al.  (2014) [63] | Handgrip | 1 | 120.0 | - | 30% MVC | |
| Greaney et al.  (2015) [64] | Handgrip | 2 | NR | 600,0 | 1^st^: 30% MVC 2^nd^: 40% MVC | |
|  |  |  |  |  |  |  |
| Grossman et al.  (1989) [65] | Handgrip | 1 | 300.0 | - | 33% MVC | |
| Hallman et al.  (2011) [66] | Handgrip | 1 | 180.0 | - | 30% MVC | |
| Heffernan et al.  (2005) [67] | Handgrip | 1 | 120.0 | - | 30% MVC | |
| Heng et al.  (1988) [68] | Handgrip | 2 | 180.0 | 300,0 | 1^st^: 20% MVC 2^nd^: 40% MVC | |
|  |  |  |  |  |  |  |
| Huikuri et al.  (1986) [71] | Handgrip | 1 | 240.0 | - | 30% MVC | |
| Ichinose et al.  (2006) [72] | Handgrip | 1 | 180.0 | - | 30% MVC | |
| Iellamo et al.  (1993) [73] | Handgrip | 3 | 120.0 | 600,0 | 30% MVC | |
|  |  |  |  |  |  |  |
|  |  |  |  |  |  |  |
| Incognito et al.  (2018) [75] | Handgrip | 1 | 120.0 | - | 30% MVC | |
| Kagaya and Homma (1997) [78] | Handgrip | 4 | 60.0 | 600 | 1^st^: 10% MVC 2^nd^: 30% MVC 3^rd^: 50% MVC 4^th^: 70% MVC | |
|  |  |  |  |  |  |  |
|  |  |  |  |  |  |  |
|  |  |  |  |  |  |  |
| Kahn et al.  (1997) [79] | Handgrip | 1  1 | 240 + 3.600  3.900.0 | - | 100% + 10% MVC  10% MVC | |
| Kalfon et al.  (2015) [80] | Handgrip | 1 | 120.0 | - | 30% MVC | |
| Kamiya et al.  (2001) [81] | Handgrip | 1 | 120.0 | - | 30% MVC | |
| Koletsos et al.  (2019) [82] | Handgrip | 1 | 180.0 | - | 30% MVC | |
| Kordi et al.  (2012) [83] | Handgrip | 1 | 180.0 | - | 30% MVC | |
| Koutnik et al.  (2014) [84] | Handgrip | 1 | 180.0 | - | 30% MVC | |
| Kramer et al.  (1983) [85] | Handgrip (unilateral and bilateral) | Right hand: 24 Left hand: 24 Both hands:  24 | 1^st^: max (NR) 2^nd^: 180.0 | - | 30% MVC | |
| Lewis et al.  (1985) [24] | Handgrip | 1 | 362.0 ± 17.2 | - | 24 ± 1% MVC | |
| Lindquist et al.  (1973) [86] | Handgrip | 1 | 90.0 | - | 50% MVC | |
| Lykidis et al.  (2008) [87] | Handgrip | 1 | 120.0 | - | 50% MVC | |
| Maiorano et al.  (1989) [88] | Handgrip | 1 | 180.0 | - | 30% MVC | |
|  |  |  |  |  |  |  |
| Majahalme et al. (1997) [89] | Handgrip | 1 | Normotensive 204.0 Borderline  228.0 Mild  186.0 | - | 30% MVC | |
| Mäkinen et al.  (2008) [90] | Handgrip | 1 | 180.0 | - | 30% MVC | |
| Matthews et al.  (2017) [91] | Handgrip | 1 | 120.0 | - | 30% MVC | |
|  |  |  |  |  |  |  |
| McCoy et al.  (1991) [92] | Handgrip | 1 | NR | - | 40% MVC | |
| McDermott et al. (1974) [93] | Handgrip | 1 | 300.0 | - | 33% MVC | |
|  |  |  |  |  |  |  |
| Metelitsina et al. (2010) [94] | Handgrip | 1 | 180.0 + 180.0 | - | NR | |
| Mizushige et al. (1997) [95] | Handgrip | 1 | 60.0 | - | 50% MVC | |
| Momen et al.  (2010) [96] | Handgrip | 2 | 20.0 | 60,0 | 1^st^: 10% MVC 2^nd^: 70% MVC | |
| Muller et al.  (2011) [98] | Handgrip | 1 | 120.0 | - | 30% MVC | |
| Nagle et al.  (1988) [99] | Handgrip | 1 | 203.4 | - | 30% MVC | |
| Notay et al.  (2018) [101] | Handgrip | 1 | 120.0 | - | 30% MVC | |
| Notay et al.  (2018b) [102] | Handgrip | 1 | 120.0 | - | 30% MVC | |
| Nyberg  (1976) [103] | Handgrip | 2 | 60.0 | NR | 50% MVC | |
| Park et al.  (2012) [104] | Handgrip | 1 | 180.0 | - | 30% MVC | |
| Parmar et al.  (2018) [105] | Handgrip | 1 | 150.0 | - | 30% MVC | |
| Pepin et al.  (1996) [106] | Handgrip | 1 | 265.8 ± 9.7 | - | 30% MVC | |
| Petrosfsky and Laymon  (2002) [107] | Handgrip | 1 | 142.0 | - | 40% MVC | |
| Piccolino et al.  (2018) [108] | Handgrip | 1 | 150.0 a 180.0 | - | 30% MVC | |
| Plotnikov et al.  (2002) [109] | Handgrip | NR | 240.0 | NR | 1^st^: 10% MVC 2^nd^: 20% MVC 3^rd^: 30% MVC | |
| Quary and Spodick (1974) [110] | Handgrip | 4 | 1^st^: 240.0 2^nd^: 240.0 3^rd^: max (NR) 4^th^: max (NR) | 900.0 | 1^st^: 15% MVC 2^nd^: 30% MVC 3^rd^: 50% MVC 4^th^: 100% MVC | |
| Sagiv et al.  (1985) [112] | Handgrip | 1 | 180.0 | - | 30% MVC | |
| Sagiv et al.  (1988c) [115] | Handgrip | 1 | 180.0 | - | 30% MVC | |
| Sagiv et al.  (1995) [116] | Handgrip | 2 | 180.0 | NR | 1^st^: 20% MVC 2^nd^: 30% MVC | |
| Samora et al.  (2019) [118] | Handgrip | 1 | 90.0 | - | 40% MVC | |
| Seals et al.  (1985) [120] | Handgrip | 2 | 1^st^: 120.0 2^nd^: 45.0 | 600.0 | 1^st^: 40% MVC 2^nd^: 60% MVC | |
| Seals et al.  (1989) [119] | Handgrip  (unilateral e bilateral) | 3 | 150.0 | 900.0 | 30% MVC | |
| Somani et al.  (2018) [28] | Handgrip | 1 | 120.0 | - | 30% MVC | |
| Stewart el al.  (2006) [121] | Handgrip | 1 | 120.0 | - | 35% MVC | |
| Tan et al.  (2013) [122] | Handgrip | 1 | 290.0 ± 17.0 | - | 35% MVC | |
| Turley et al.  (2005) [124] | Handgrip | 1 | 180.0 | - | 30% MVC | |
| Umeda et al.  (2009) [125] | Handgrip | 1 | 60.0/ 180.0 (random) | - | 25% MVC | |
| Umeda et al.  (2015) [126] | Handgrip | 1 | 180.0 | - | 25% MVC | |
| Van Huysduynen et al.  (2004) [127] | Handgrip | 1 | 180.0 | - | 30% MVC | |
| Vaz et al.  (1993) [128] | Handgrip | 1 | 180.0 | - | 30% MVC | |
| Vianna et al.  (2012) [129] | Handgrip | 1 | 120.0 | - | 30% MVC | |
| Williams  (1991) [132] | Handgrip | 2 | 1^st^: 46.0 ± 3.3 2^nd^: 60.0 | NR | 1^st^: 70% MVC 2^nd^: 100% MVC | |
| Fujisawa et al.  (1996) [56] | One-knee extension | 1 | 58.1 ± 9.3 | - | 60% MVC | |
| Hickey et al.  (1993) [69] | Two-knee extension | 1 | 178.5 ± 17.2 178.8 ± 11.7 | - | 30% MVC | |
| Hirasawa et al.  (2016) [70] | One-knee extension | 1 | 120.0 | - | 30% MVC | |
| Iellamo et al.  (1999) [74] | One-knee extension | 1 | 240.0 | - | 30% MVC | |
| Kadetoff and Kosek (2007) [76] | One-knee extension | 1 | 600.0 | - | 10% MVC | |
| Kadetoff and Kosek (2010) [77] | Two-knee extension | 1 | 960.0 | - | 8% MVC | |
| Lewis et al.  (1985) [24] | Two-knee extension | 1 | 324.0 ± 9.8 | - | 25 ± 2.5 % MVC | |
| Nagle et al.  (1988) [99] | Two-knee extension | 1 | 216.6 | - | 30% MVC | |
| Petrosfsky and Laymon (2002) [107] | Two-knee extension | 1 | 120.0 | - | 40% MVC | |
| Seals et al.  (1983) [23] | One-knee extension | 1 | NR | - | 30% MVC | |
| Somani et al.  (2018) [28] | Two-knee extension | 1 | 120.0 | - | 20% MVC | |
| Williams  (1991) [132] | Two-knee extension | 2 | 1^st^: 53.0 ± 3.4 2^nd^: 60.0 | NR | 1^st^: 70% MVC 2^nd^: 100% MVC | |
| Wright et al.  (1999) [133] | One-knee extension | 1 | 180.0 | - | 30% MVC | |
| Yamaji et al.  (1983) [25] | One-knee extension | 5 | 1^st^: 540.0 a 600.0 2^nd^: 180.0 a 240.0 3^rd^: 120.0 a 180.0 4^th^: 60.0 a 120.0 5^th^: 60.0 a 120.0 | - | 1^st^: 10% MVC 2^nd^: 20% MVC 3^rd^: 30% MVC 4^th^: 40% MVC 5^th^: 50% MVC | |
| Nagle et al.  (1988) [99] | Deadlift | 1 | 220.8 | - | 30% MVC | |
| Sagiv et al.  (1985) [112] | Deadlift | 1 | 180.0 | - | 30% MVC | |
| Sagiv et al.  (1988) [113] | Deadlift | 1 | 180.0 | - | 30% MVC | |
| Sagiv et al.  (1988b) [114] | Deadlift | 1 | 180.0 | - | 30% MVC | |
| Sagiv et al.  (1988c) [115] | Deadlift | 1 | 180.0 | - | 30% MVC | |
| Sagiv et al.  (1995) [116] | Deadlift | 2 | 180.0 | NR | 1^st^: 20% MVC 2^nd^: 30% MVC | |
| Sagiv et al.  (2008) [117] | Deadlift | 1 | 180.0 | - | 30% MVC | |
| Vitcenda et al.  (1990) [130] | Deadlift | 1 | 32.0 | - | 90% MVC | |
| Davies and Starkie (1985) [49] | Elbow flexion | 1 | 120.0 | 1.800,0 | 30% MVC | |
| Mortensen et al.  (2016) [97] | Elbow flexion(unilateral) | 1 | 180.0 | - | 35% MVC | |
| Nakamura et al.  (2005) [100] | Elbow flexion(unilateral) | 1 | 120.0 | - | 35% MVC | |
| Yamaji et al.  (1983) [22] | Elbow flexion | 5 | 1^st^: 720.0 a 780.0 2^nd^: 360.0 a 420.0 3^rd^: 180.0 a 240.0 4^th^: 120.0 a 180.0 5^th^: 60.0 a 120.0 | - | 1^st^: 10% MVC 2^nd^: 20% MVC 3^rd^: 30% MVC 4^th^: 40% MVC 5^th^: 50% MVC | |
| Dias and Polito  (2015) [48] | Squat | 6 | 30.0 | 30.0 | 20% RM | |
| Taylor et al.  (2017) [121] | Wall squat | 4 | 120.0 | 120.0 | Knee angulation corresponding to 95% of peak HR achieved in the incremental test | |
| Wiles et al.  (2018) [15] | Wall squat | 4 | 120.0 | 120.0 | Knee angulation corresponding to 95% of peak HR achieved in the incremental test | |
| Da Silva et al.  (2013) [23] | Leg press (45°) | HI: 3 MI: 3 LI: 3 | HI: 30.0 MI: 60.0 LI: 30.0 | HI: 60.0 MI: 60.0 LI: 60.0 | HI: 60% MVC MI: 20% MVC LI: 20% MVC | |
| Weippert et al.  (2013) [129] | Leg press | 1 | 300.0 | - | 20 kg | |
| Davies and Starkie (1985) [47] | Plantar flexion | 2 | 1^st^: 120.0 2^nd^: 60.0 | 1.800.0 | 1^st^: 30% MVC 2^nd^: 50% MVC | |
| Riendl et al.  (1977) [109] | Plantar flexion | 3 | 1^st^: 516.0 ± 96.0 2^nd^: 432.0 ± 72.0 3^rd^: 378.0 ± 48.0 | 300.0 | 30% MVC | |
| Seals et al.  (1983) [20] | Elbow extension | 1 | Max (NR) | - | 30% MVC | |
| Auerbach et al.  (2000) [38] | Whole-body isometric exercise | 2 | 180.0 | 600,0 | 1^st^: 30% MVC 2^nd^: 50% MVC | |
| Plotnikov et al.  (2002) [107] | Torso effort | NR | 240.0 | NR | 1^st^: 10% MVC 2^nd^: 20% MVC 3^rd^: 30% MVC | |
| Ben-Ari et al.  (1992) [42] | Two-hand pulling | 1 | 120.0 | - | 50% MVC | |
| Riendl et al.  (1977) [109] | Finger adduction | 3 | 1^st^: 684.0 ± 240.0 2^nd^: 1.008.0 ± 276.0 3^rd^: 948.0 ± 300.0 | 300,0 | 30% MVC | |

Note: Data presented as mean ± standard deviation. MVC= maximum voluntary contraction. RM= one-repetition maximum. HR= heart rate. AI= high intensity. MI= moderate intensity. BI= low intensity. NR= not reported.
