# Supplementary material for: Acute Blood Pressure Response to Different Types of Isometric Exercise: A Systematic Review with Meta-Analysis
Source: Rev Cardiovasc Med. 2023 Feb 10;24(2):60. doi: 10.31083/j.rcm2402060 (PMC11273125; doi:10.31083/j.rcm2402060)
Supplement: Supplementary file 1 [file 2153-8174-24-2-060-s1.zip › Supplementary material 3.docx]

| **Blood pressure responses during isometric handgrip exercise.** | | | | | | | | |
| --- | --- | --- | --- | --- | --- | --- | --- | --- |
| **Author and year** | **Pre SBP** | **During SBP** | **Δ SBP** | **Mean %Δ** | **Pre DBP** | **During DBP** | **Δ DBP** | **Mean %Δ** |
|  |  |  |  | ***Handgrip*** |  |  |  |  |
| Almeida et al. (2021) [10] | Offspring of NT  121.00 ± 8.23 Offspring of HT  124.40 ± 11.60 | Offspring of NT  131.90 ± 15.34 Offspring of HT  137.70 ± 17.21 | Offspring of NT  +10.90 Offspring of HT  +13.30 | Offspring of NT  9.01 Offspring of HT  10.69 | Offspring of NT  64.70 ± 6.36 Offspring of HT  65.50 ± 5.61 | Offspring of NT  75.70 ± 8.98 Offspring of HT  78.20 ± 13.84 | Offspring of NT  +11.00 Offspring of HT  +12.70 | Offspring of NT 17.00  Offspring of HT 19.39 |
| Aoki et al. (1983) [40] | Normotensive  112.90 ± 7.20 Hypertensive  139.60 ± 15.30 | Normotensive  142.70 ± 8.80 Hypertensive  184.10 ± 19.10 | Normotensive  +29.80 ± 8.50 Hypertensive  +44.50 ± 14.10 | Normotensive  26.40 Hypertensive  31.88 | Normotensive  69.70 ± 6.70 Hypertensive  93.00 ± 10.90 | Normotensive  96.10 ± 7.60 Hypertensive  122.20 ± 13.90 | Normotensive  +26.40 ± 7.20 Hypertensive  +29.20 ± 9.10 | Normotensive 37.88 Hypertensive  31.40 |
| Bakke et al. (2007) [42] | Young  120.80 ± 13.50 Elderly  130.60 ± 1.10 | Young  147.20 ± 12.50 Elderly  177.40 ± 0.70 | Young  +26.40 ± 12.30 Elderly  +40.00 ± 21.10 | Young  21.85 Elderly  30.63 | Young  68.40 ± 6.80 Elderly  63.40 ± 0.50 | Young  89.80 ± 9.30 Elderly  89.10 ± 0.30 | Young  +21.40 ± 8.40 Elderly  +25.80 ± 7.00 | Young  31.29 Elderly  40.69 |
| Bakke et al. (2009) [43] | 124.70 ± 17.70 | 169.00 ± 24.60 | +44.30 | 35.53 | 64.60 ± 8.70 | 95.70 ± 15.90 | +31.10 | 48.14 |
| Balmain et al. (2016) [44] | 126.00 ± 26.15 | NR | +35.00 ± 13.08 | 27.78 | 74.00 ± 21.79 | NR | +28.00 ± 8.72 | 37.84 |
| Bentley and Thomas (2018) [9] | 109.10 ± 9.10 | 145.00 ± 20.30 | +35.70 ± 2.50 | 32.72 | 73.30 ± 7.70 | 89.60 ± 9.30 | +15.90 ± 1.00 | 21.69 |
| Borghi et al. (1988) [46] | Offspring of NT  107.6 ± 8.0 Offspring of HT  108.7 ± 5.0 | NR | Offspring of NT  +16.1 ± 6.0 Filhos de HT  +24.0 ± 3.0 | Offspring of NT  14.96  Offspring of HT  22.08 | Offspring of NT  60.6 ± 5.0 Offspring of HT  63.5 ± 6.0 | NR | Offspring of NT  +11.9 ± 2.0 Offspring of HT  +18.0 ± 2.0 | Offspring of NT  19.63 Offspring of HT  28.35 |
| Bosisio et al. (1980) [47] | Upper limbs  117.50 ± 10.69  Lower limbs  128.70 ± 15.06 | 1^st^ - Upper limbs  138.10 ± 16.89 Lower limbs  160.60 ± 38.77 2^nd^ - Upper limbs  146.20 ± 14.82 Lower limbs  159.38 ± 34.38 | 1^st^ - Upper limbs  +20.60 Lower limbs  +31.90  2^nd^ - Upper limbs  +28.70 Lower limbs  +30.68 | 1^st^ - Upper limbs  17.53 Lower limbs  24.79 2^nd^ - Upper limbs  24.43 Lower limbs  23.84 | NR | NR | NR | _ |
| Cottone et al. (1998) [48] | Normotensive  124.00 ± 20.78 Hypertensive  161.00 ± 19.36 | Normotensive  145.00 ± 10.39 Hypertensive  188.00 ± 7.75 | Normotensive  +21.00 Hypertensive  +27.00 | Normotensive  16.93 Hypertensive  16.77 | Normotensive  65.00 ± 13.86 Hypertensive  93.00 ± 7.75 | Normotensive  84.00 ± 13.86 Hypertensive  110.00 ± 11.62 | Normotensive  +19.00 Hypertensive  +17.00 | Normotensive  29.23 Hypertensive  18.28 |
|  |  |  |  |  |  |  |  |  |
| Ehsani et al. (1981) [51] | 114.00 ± 12.72 | 1^st^: 130.60 ± 14.97 2^nd^ :138.60 ± 14.97 3^rd^: 158.50 ± 16.46 | 1^st^: +16.60 2^nd^: +24.60 3^rd^: +44.50 | 1^st^: 14.56 2^nd^: 21.58 3^rd^: 39.04 | 72.90 ± 11.22 | 1^st^: 87.80 ± 11.22 2^nd^: 97.00 ± 13.47 3^rd^: 111.70 ± 10.85 | 1^st^: +14.90 2^nd^: +24.10 3^rd^: +38.80 | 1^st^: 20.44 2^nd^: 33.06 3^rd^: 53.22 |
|  |  |  |  |  |  |  |  |  |
|  |  |  |  |  |  |  |  |  |
| Ehsani et al. (1982) [52] | 127.00 ± 13.86 | 1^st^: 151.00 ± 13.86 2^nd^: 175.00 ± 13.86 | 1^st^: +24.00 2^nd^: +48.00 | 1^st^: 18.90 2^nd^: 37.80 | 81.00 ± 6.93 | 1^st^: 104.00 ± 13.86 2^nd^: 123.00 ± 13.86 | 1^st^: +23.00 2^nd^: +42.00 | 1^st^: 28.40 2^nd^: 51.85 |
|  |  |  |  |  |  |  |  |  |
| Ferguson and Brown (1997) [53] | Athletes  124.00 ± 6.71 Sedentary  136.00 ± 18.97 | Athletes  149.00 ± 17.89 Sedentary  184.00 ± 25.30 | Athletes  +25.00 ± 29.07 Sedentary  +48.00 ± 53.76 | Athletes  20.16 Sedentary  35.29 | Athletes  72.00 ± 13.42 Sedentary  72.00 ± 12.65 | Athletes  101.00 ± 11.18 Sedentary  108.00 ± 18.97 | Athletes  +29.00 Sedentary  +36.00 | Athletes  40.28 Sedentary  50.00 |
| Fu et al. (1981) [54] | Normotensive  121.80 ± 8.05 Hypertensive  154.00 ± 17.75 | Normotensive  145.30 ± 11.63 Hypertensive  188.70 ± 15.21 | Normotensive  +23.50 Hypertensive  +34.70 | Normotensive  19.29 Hypertensive  22.53 | Normotensive  75.40 ± 7.16 Hypertensive  91.90 ± 11.83 | Normotensive  93.80 ± 4.92 Hypertensive  114.80 ± 12.42 | Normotensive  +18.40 Hypertensive  +22.90 | Normotensive  24.40 Hypertensive  24.92 |
|  |  |  |  |  |  |  |  |  |
| Fu et al. (2002) [55] | 141.00 ± 13.42 | 186.00 ± 24.60 | +45.00 | 31.91 | 73.00 ± 11.18 | 99.00 ± 11.18 | +26.00 | 35.62 |
| Gois et al. (2019) [57] | Supine  123.60 ± 12.50 Sitting  127.20 ± 16.50 Standing  128.00 ± 12.20 | Supine  176.90 ± 25.00 Sitting  185.90 ± 23.90 Standing  192.20 ± 22.50 | Supine  +53.30 Sitting  +8.70 Standing  +64.20 | Supine  43.12 Sitting  46.15 Standing  50.16 | Supine  68.60 ± 6.70 Sitting  77.00 ± 9.50 Standing  78.20 ± 7.20 | Supine  98.90 ± 14.00 Sitting  105.40 ± 15.40 Standing  109.40 ± 12.70 | Supine  +30.30 Sitting  +28.40 Standing  +31.20 | Supine  44.17 Sitting  36.88 Standing  39.90 |
| Goldstein and Shapiro (1988) [58] | Sitting  121.30 ± 10.90 Standing  117.20 ± 13.50 | Sitting  126.60 ± 12.50 Standing  126.10 ± 12.70 | Sitting  +5.30 Standing  +8.90 | Sitting  4.37 Standing  7.59 | Sitting  73.80 ± 11.10 Standing  77.80 ± 11.60 | Sitting  78.60 ± 10.90 Standing  84.20 ± 10.10 | Sitting  +4.80 Standing  +6.40 | Sitting  6.50 Standing  8.23 |
| Goldstraw and Warren (1985) [59] | Young  116.00 ± 7.97 Elderly  134.00 ± 6.58 | Young  1^st^: 122.00 ± 7.27 2^nd^: 135.00 ± 11.43 3^rd^: 148.00 ± 10.74 Elderly  1^st^: 146.000 ± 7.27 2^nd^: 164.00 ± 16.28 3^rd^: 173.00 ± 21.82 | Young  1^st^: +6.00 2^nd^: +19.00 3^rd^: +32.00 Elderly  1^st^: +12.00 2^nd^: +30.00 3^rd^: +39.00 | Young  1^st^: 5.17 2^nd^: 16.38 3^rd^: 27.59 Elderly  1^st^: 8.96 2^nd^: 22.39 3^rd^: 29.10 | Young  66.00 ± 6.24 Elderly  72.00 ± 6.24 | Young  1^st^: 70.00 ± 6.24 2^nd^: 82.00 ± 8.66 3^rd^: 91.00 ± 8.31 Elderly  1^st^: 78.00 ± 7.27 2^nd^: 90.00 ± 9.01 3^rd^: 98.00 ± 8.66 | Young  1^st^: +4.00 2^nd^: +16.00 3^rd^: +25.00 Elderly  1^st^: +6.00 2^nd^: +18.00 3^rd^: +26.00 | Young  1^st^: 6.06 2^nd^: 24.24 3^rd^: 37.88 Elderly  1^st^: 8.33 2^nd^: 25.00 3^rd^: 36.11 |
| Goulopoulou et al. (2010) [60] | 117.50 ± 10.55 | 148.60 ± 17.74 | +31.10 | 26.47 | 71.30 ± 5.75 | 91.90 ± 10.07 | +20.60 | 28.89 |
| Graafsma et al. (1989) [61] | Normotensive  112.00 ± 9.49 Hypertensive  141.00 ± 21.63 | Normotensive  140.00 ± 18.97 Hypertensive  164.00 ± 28.84 | Normotensive  +28.00 Hypertensive  +23.00 | Normotensive  25.00 Hypertensive  16.31 | Normotensive  78.00 ± 6.32 Hypertensive  97.00 ± 7.21 | Normotensive  107.00 ± 15.81 Hypertensive  115.00 ± 10.82 | Normotensive  +29.00 Hypertensive  +18.00 | Normotensive  37.18 Hypertensive 18.56 |
|  |  |  |  |  |  |  |  |  |
| Greaney et al. (2013) [62] | Young  134.00 ± 15.81 Middle-aged/elderly 137.00 ± 18.00 | Young  159.00 ± 22.14 Middle-aged/elderly 166.00 ± 18.00 | Young  +25.00 Middle-aged/elderly +29.00 | Young  18.66 Middle-aged/elderly 21.17 | Young  69.00 ± 3.16 Middle-aged/elderly  71.00 ± 9.00 | Young  83.00 ± 6.32 Middle-aged/elderly 87.00 ± 9.00 | Young  +14.00 Middle-aged/elderly +16.00 | Young  20.29 Middle-aged/elderly  22.54 |
| Greaney et al. (2014) [63] | Young  112.00 ± 6.63 Elderly  117.00 ± 6.93 | NR | Young  +29.00 ± 13.27 Elderly  +24.00 ± 17.32 | Young  25.89 Elderly  20.51 | Young  71.00 ± 6.63 Elderly  70.00 ± 6.93 | NR | Young  +17.00 ± 6.63 Elderly  +15.00 ± 10.39 | Young  23.94 Elderly  21.43 |
| Greaney et al. (2015) [64] | Normotensive  112.00 ± 4.80 Hypertensive  153.00 ± 11.62 | NR | Normotensive  1^st^: +10.00 ± 9.59 2^nd^: +11.00 ± 9.59 Hypertensive  1^st^: +19.00 ± 15.49 2^nd^: +14.00 ± 15.49 | Normotensive  1^st^: 8.93  2^nd^: 9.82 Hypertensive  1^st^: 12.42 2^nd^: 9.15 | Normotensive  67.00 ± 9.59 Hypertensive  91. 00 ± 19.36 | NR | Normotensive  1^st^: + 6.00 ± 4.80 2^nd^: + 6.00 ± 4.80 Hypertensive  1^st^: + 9.00 ± 7.75 2^nd^: + 13.00 ± 7.75 | Normotensive  1^st^: 8.96 2^nd^: 8.96 Hypertensive  1^st^: 9.89 2^nd^: 14.29 |
|  |  |  |  |  |  |  |  |  |
|  |  |  |  |  |  |  |  |  |
|  |  |  |  |  |  |  |  |  |
| Grossman et al. (1989) [65] | 159.00 ± 18.00 | 200.00 ± 27.00 | +41.00 | 25.79 | 88.00 ± 8.00 | 112.00 ± 14.00 | +24.00 | 27.27 |
| Hallman et al. (2011) [66] | 123.00 ± 12.00 | 150.00 ± 15.00 | +27.00 | 21.95 | 62.00 ± 8.00 | 81.00 ± 10.00 | +19.00 | 30.65 |
| Heffernan et al. (2005) [67] | 116.50 ± 12.33 | 150.10 ± 16.76 | +33.60 | 28.84 | 56.20 ± 10.12 | NR | NR | _ |
| Heng et al. (1988) [68] | 113.00 ± 10.00 | 1^st^: 134.00 ± 9.00 2ª: 163.00 ± 7.00 | 1^st^: +21.00 2ª: +50.00 | 1^st^: 18.58 2ª: 44.25 | NR | NR | NR | _ |
|  |  |  |  |  |  |  |  |  |
| Huikuri et al. (1986) [71] | 115.00 ± 11.46 | 136.00 ± 16.21 | +21.00 | 18.26 | 70.00 ± 9.26 | 91.00 ± 12.97 | +21.00 | 30.00 |
| Ichinose et al. (2006) [72] | 126.00 ± 11.90 | 161.00 ± 11.18 | +35.00 | 27.78 | 67.00 ± 7.21 | 92.00 ± 7.93 | +25.00 | 37.31 |
| Iellamo et al. (1993) [73] | Supine  122.60 ± 12.33 Sitting  120.80 ± 6.32 Standing  122.00 ± 10.44 | NR | Supine  +21.40 ± 8.85 Sitting  +24.00 ± 5.38 Standing  +24.50 ± 7.91 | Supine  17.46 Sitting  19.87 Standing  20.08 | Supine  60.20 ± 9.49 Sitting  72.30 ± 5.69 Standing  74.20 ± 6.32 | NR | Supine  +13.50 ± 7.91 Sitting  +3.90 ± 3.48 Standing  +13.20 ± 6.96 | Supine  22.43 Sitting  9.23 Standing  17.79 |
|  |  |  |  |  |  |  |  |  |
|  |  |  |  |  |  |  |  |  |
| Incognito et al. (2018) [75] | 109.00 ± 7.00 | 126.00 ± 14.00 | 17.00 | 15.60 | 64.00 ± 6.00 | 78.00 ± 9.00 | 14.00 | 21.88 |
| Kagaya and Homma (1997) [78] | 120.90 ± 10.05 | NR | 1^st^: +16.10 ± 9.07 2^nd^: +24.20 ± 10.85 3^rd^: +33.80 ± 17.73 4^th^: +46.10 ± 20.90 | 1^st^: 13.32 2^nd^: 20.02 3^rd^: 27.96 4^th^: 38.13 | 67.00 ± 3.97 | NR | 1^st^: +9.00 ± 4.50 2^nd^: +17.50 ± 7.41 3^rd^: +25.00 ± 12.96 4^th^: +42.90 ± 23.55 | 1^st^: 13.43 2^nd^: 26.12 3^rd^: 37.31 4^th^: 64.03 |
|  |  |  |  |  |  |  |  |  |
|  |  |  |  |  |  |  |  |  |
|  |  |  |  |  |  |  |  |  |
| Kahn et al. (1997) [79] | 117.50 ± 8.66 112.90 ± 6.93 | 178.60 ± 15.59 139.50 ± 11.78 | +61.10 +26.60 | 52.00 23.56 | NR | NR | NR | _ |
|  |  |  |  |  |  |  |  |  |
| Kalfon et al. (2015) [80] | HG1  123.00 ± 12.00 HG2  122.00 ± 8.00 | HG1  143.00 ± 16.00 HG2  140.00 ± 12.00 | HG1  +20.00 HG2  +18.00 | HG1  16.26 HG2  14.75 | HG1  68.00 ± 8.00 HG2  67.00 ± 4.00 | HG1  76.00 ± 12.00 HG2  76.00 ± 8.00 | HG1  +8.00 HG2  +9.00 | HG1  11.76 HG2  13.43 |
| Kamiya et al. (2001) [81] | 120.30 ± 17.35 | 139.80 ± 23.45 | +19.50 | 16.21 | 74.30 ± 11.73 | 89.20 ± 20.17 | +14.90 | 20.05 |
| Koletsos et al. (2018) [82] | Normotensive  127.40 ± 11.10 Masked HT  134.20 ± 9.70 True HT  143.60 ± 13.30 | Normotensive  159.60 ± 15.90 Masked HT  174.80 ± 13.50 True HT  179.30 ± 16.10 | Normotensive  +32.20 Masked HT  +40.60 True HT  +35.70 | Normotensive  25.27 Masked HT  30.25 True HT  24.86 | Normotensive  73.70 ± 7.99 Masked HT  77.90 ± 7.20 True HT  84.30 ± 7.80 | Normotensive  91.40 ± 9.40 Masked HT  98.50 ± 7.00 True HT  100.80 ± 10.60 | Normotensive  +17.70 Masked HT  +20.60 True HT  +16.50 | Normotensive  24.02 Masked HT  26.44 True HT  19.57 |
|  |  |  |  |  |  |  |  |  |
|  |  |  |  |  |  |  |  |  |
| Kordi et al. (2012) [83] | 116.60 ± 12.34 | NR | +11.45 ± NR | 9.82 | 66.60 ± 10.30 | NR | +14.05 ± NR | 21.10 |
| Koutnik et al. (2014) [84] | 116.00 ± 8.94 | 147.00 ± 22.36 | +31.00 | 26.72 | 59.00 ± 13.42 | 86.00 ± 17.89 | +27.00 | 45.76 |
| Kramer et al. (1983) [85] | Right  188.87 ± 4.73 Left  183.77 ± 4.43 Bilateral  184.97 ± 4.88 | Right  227.12 ± 8.25 Left  220.29 ± 5.85 Bilateral  237.92 ± 10.65 | Right  +38.25 Left  +36.52 Bilateral  +52.95 | Right  20.25 Left  19.87 Bilateral  28.63 | Right  96.38 ± 5.55 Left  98.78 ± 4.13 Bilateral  100.73 ± 5.40 | Right  118.51 ± 6.00 Left  117.23 ± 5.78 Bilateral  126.99 ± 6.83 | Right  +22.13 Left  +18.45 Bilateral  +26.26 | Right  22.96 Left  18.68 Bilateral  26.07 |
| Lewis et al. (1985) [24] | 118.00 ± 7.35 | 150.00 ± 14.70 | +32.00 | 27.12 | 67.00 ± 7.35 | 94.00 ± 9.80 | +27.00 | 40.30 |
| Lindquist et al. (1973) [86] | 120.00 ± 13.00 | 165.00 ± 20.00 | +45.00 | 37.50 | 73.00 ± 10.00 | 108.00 ± 15.00 | +35.00 | 47.95 |
| Lykidis et al. (2008) [87] | 136.43 ± 15.09 | 163.69 ± 20.16 | +27.26 | 19.98 | 71.59 ± 11.22 | 93.48 ± 15.72 | +21.89 | 30.58 |
| Maiorano et al. (1989) [88] | Trained  122.26 ± 14.70 Untrained  125.20 ± 12.10 | Trained  146.24 ± 18.00 Untrained  152.64 ± 16.90 | Trained  +23.98 Untrained  +27.44 | Trained  19.61 Untrained  21.92 | Trained  65.90 ± 10.80 Untrained  69.92 ± 10.30 | Trained  82.27 ± 18.00 Untrained  88.83 ± 22.00 | Trained  +16.37 Untrained  +18.91 | Trained  24.84 Untrained  27.05 |
|  |  |  |  |  |  |  |  |  |
| Majahalme et al. (1997) [89] | Normotensive  123.8 ± 7.50 Borderline HT  137.4 ± 7.30 Mildly HT  141.2 ± 7.30 | Normotensive  171.0 ± 17.7 Borderline HT  185.9 ± 14.30 Mildly HT  192.8 ± 19.10 | Normotensive  +47.0 ± 2.80 Borderline HT  +51.9 ± 4.00 Mildly HT  +48.2 ± 3.10 | Normotensive  37.96 Borderline HT  37.77 Mildly HT  34.14 | Normotensive  75.10 ± 5.40 Borderline HT  84.30 ± 4.70 Mildly HT  90.30 ± 7.90 | Normotensive  103.70 ± 7.90 Borderline HT  115.00 ± 11.40 Mildly HT  121.90 ± 11.10 | Normotensive  +32.70 ± 1.70 Borderline HT  +35.5 ± 2.50 Mildly HT  +36.5 ± 1.70 | Normotensive  43.54 Borderline HT 42.11 Mildly HT  40.42 |
| Makinen et al. (2008) [90] | 131.00 ± 5.00 | 150.00 ± 5.00 | +19.00 | 14.50 | 79.00 ± 6.00 | 101.00 ± 8.00 | +22.00 | 27.85 |
| Matthews et al. (2017) [91] | FH-  127.00 ± 19.00 FH+  122.00 ± 17.00 | FH-  130.00 ± 20.00 FH+  133.00 ± 17.00 | FH-  +3.00  FH+  +11.00 | FH-  2.36  FH+  9.02 | FH-  64.00 ± 11.00 FH+  61.00 ± 13.00 | FH-  67.00 ± 12.00 FH+  70.00 ± 13.00 | FH-  +3.00 FH+  +9.00 | FH-  4.69 FH+  14.75 |
|  |  |  |  |  |  |  |  |  |
| McCoy et al. (1991) [92] | 132.76 ± 11.25 | 170.26 ± 24.75 | +36.75 ± 11.25 | 27.68 | 81.76 ± 6.75 | 111.76 ± 15.75 | +30.75 ± 9.00 | 37.61 |
| McDermott et al. (1974) [93] | Young  128.00 ± 7.27 Middle-aged  117.20 ± 11.09 | Young  191.20 ± 18.66 Middle-aged  191.40 ± 18.71 | Young  +63.20 Middle-aged  +74.20 | Young  49.38 Middle-aged  63.31 | Young  86.70 ± 8.85 Middle-aged  82.80 ± 8.31 | Young  137.50 ± 13.60 Middle-aged  142.20 ± 16.63 | Young  +50.80 Middle-aged  +59.40 | Young  58.59 Middle-aged  71.74 |
|  |  |  |  |  |  |  |  |  |
| Metelitsina et al. (2010) [94] | 130.30 ± 15.69 | NR | +35.70 ± 21.49 | 27.40 | 75.60 ± 10.90 | NR | +20.20 ± 22.75 | 26.72 |
| Mizushige et al. (1997) [95] | 123.00 ± 18.00 | 155.00 ± 17.00 | +32.00 | 26.02 | 76.00 ± 7.00 | 99.00 ± 6.00 | +23.00 | 30.26 |
| Momen et al. (2010) [96] | NR | NR | NR | _ | Men  71.5 ± 8.62 Women  76.4 ± 6.30 | NR | Men  1^st^: + 0.8 ± 2.32 2^nd^: + 12.4 ± 9.29 Women  1^st^: + 1.6 ± 4.64 2^nd^: + 14.2 ± 7.96 | Men  1^st^: 1.12 2^nd^: 17.34 Women  1^st^: 2.09 2^nd^: 18.59 |
|  |  |  |  |  |  |  |  |  |
|  |  |  |  |  |  |  |  |  |
|  |  |  |  |  |  |  |  |  |
| Muller et al. (2011) [98] | 101.00 ± 6.32 | 128.00 ± 12.65 | +27.00 | 26.73 | 57.00 ± 9.49 | 77.00 ± 12.65 | +20.00 | 35.09 |
| Nagle et al. (1988) [99] | 123.00 ± 8.00 | 143.00 ± 12.00 | +20.00 | 16.26 | 79.00 ± 4.00 | 94.00 ± 6.00 | +15.00 | 18.99 |
| Notay et al. (2018) [101] | Men  107.00 ± 8.00 Women  102.00 ± 9.00 | NR | Men  +26.04 ± 11.32 Women  +18.98 ± 10.19 | Men  24.34 Women  18.61 | Men  64.00 ± 7.00 Women  66.00 ± 8.00 | NR | Men  +19.10 ± 7.62 Women  +15.61 ± 6.95 | Men  29.84 Women  23.65 |
| Notay et al. (2018b) [102] | 104.00 ± 8.00 | NR | +20.00 ± 10.00 | 19.23 | 66.00 ± 7.00 | NR | +16.00 ± 7.00 | 24.24 |
| Nyberg (1976) [103] | NT Men  125.00 ± 13.31  NT Women  108.00 ± 10.71  HT Men  (non-treated)  157.00 ± 24.30  HT Women  (non-treated)  149.00 ± 15.66  HT Men  (treated)  143.00 ± 19.47  HT Women  (treated)  149.00 ± 32.49 | NT Men  154.00 ± 12.55  NT Women  135.00 ± 23.79  HT Men  (non-treated)  196.00 ± 30.69  HT Women  (non-treated)  193.00 ± 30.55  HT Men  (treated)  180.00 ± 25.05  HT Women  (treated)  190.00 ± 40.67 | NT Men  +30.00  NT Women  +26.00  HT Men  (non-treated)  +39.00  HT Women  (non-treated)  +44.00  HT Men  (treated)  +36.00  HT Women  (treated)  +41.00 | NT Men  24.00  NT Women  24.07  HT Men  (non-treated)  24.84  HT Women  (non-treated)  29.53  HT Men  (treated)  25.17  HT Women  (treated)  27.52 | NT Men  80.00 ± 8.73  NT Women  72.00 ± 8.49  HT Men  (non-treated)  97.00 ± 12.54  HT Women  (non-treated)  99.00 ± 9.73  HT Men  (treated)  92.00 ± 12.68  HT Women (treated)  93.00 ± 8.59 | NT Men  109.00 ± 14.01  NT Women  104.00 ± 18.39  HT Men  (non-treated)  127.00 ± 16.29  HT Women  (non-treated)  123.00 ± 15.42  HT Men  (treated)  117.00 ± 10.88  HT Women  (treated)  122.00 ± 12.57 | NT Men  +29.00  NT Women  +32.00  HT Men  (non-treated)  +30.00  HT Women  (non-treated)  +24 .00  HT Men  (treated)  +25.00  HT Women (treated)  +29.00 | NT Men  36.25  NT Women  44.44  HT Men  (non-treated)  30.93  HT Women  (non-treated)  24.24  HT Men  (treated)  27.17  HT Women (treated)  31.18 |
| Park et al. (2012) [104] | Lean  117.80 ± 10.74 Overweight  124.40 ± 8.31 | NR | Lean  +22.40 ± 12.12 Overweight  +16.60 ± 5.54 | Lean  19.02 Overweight  13.34 | Lean  69.30 ± 10.05 Overweight  72.30 ± 8.31 | NR | Lean  _+16.60 ± 7.97  Overweight  +14.80 ± 8.66 | Lean  23.95 Overweight  20.47 |
| Parmar et al. (2018) [105] | Men  114.00 ± 6.63 Women NC  103.00 ± 9.00 Women OC  109.00 ± 3.16 | Men  153.00 ± 19.90 Women NC  129.00 ± 15.00 Women OC  145.00 ± 15.81 | Men  +39.00 Women NC  +26.00 Women OC  +36.00 | Men  34.21 Women NC  25.24 Women OC  33.03 | Men  66.00 ± 3.32 Women NC  61.00 ± 6.00 Women OC  67.00 ± 6.32 | Men  94.00 ± 9.95 Women NC  80.00 ± 12.00 Women OC  93.00 ± 6.32 | Men  +28.00 Wome NC  +19.00 Women OC +26.00 | Men  42.42 WomenNC  31.15 Women OC  38.81 |
|  |  |  |  |  |  |  |  |  |
|  |  |  |  |  |  |  |  |  |
| Pepin et al. (1996) [106] | 143.10 ± 14.10 | NR | +58.30 ± 19.50 | 40.74 | 76.80 ± 9.30 | NR | +39.80 ± 9.50 | 51.82 |
| Petrosfsky and Laymon (2002) [107] | 20-30 years  120.76 ± 9.00 31-40 years  123.01 ± 8.30 41-50 years  129.01 ± 9.00 51-65 years  135.01 ± 8.30 | 20-30 years  180.77 ± 2.30 31-40 years  185.27 ± 9.00 41-50 years  189.02 ± 12.80 51-65 years  198.77 ± 14.30 | 20-30 years  +60.01 31-40 years  +62.26 41-50 years  +60.01 51-65 years  +63.76 | 20-30 years  49.69 31-40 years  50.61 41-50 years  46.52 51-65 years  47.23 | 20-30 years  78.00 ± 11.30 31-40 years  76.50 ± 9.00 41-50 years  83.25 ± 12.00 51-65 years  84.75 ± 8.30 | 20-30 years  132.01 ± 15.00 31-40 years  137.26 ± 12.00 41-50 years  141.01 ± 9.00 51-65 years  144.01 ± 14.30 | 20-30 years  +54.01 31-40 years  +60.76 41-50 years  +57.76 51-65 years  +59.26 | 20-30 years  69.24 31-40 years  79.42 41-50 years  69.38 51-65 years  69.92 |
| Piccolino et al. (2018) [108] | 112.20 ± 7.70 | 143.44 ± 10.95 | +31.24 ± 9.21 | 27.84 | 75.12 ± 5.35 | 97.24 ± 7.12 | +22.12 ± 6.00 | 29.45 |
| Plotnikov et al. (2002) [109] | 108.00 ± 15.93 | 1^st^: 107.50 ± 17.32 2^nd^: 118.00 ± 18.01 3^rd^: 133.30 ± 13.86 | 1^st^: -0.50 2^nd^: +10.00 3^rd^: +25.30 | 1^st^: -0.46 2^nd^: 9.26 3^rd^: 23.43 | 68.30 ± 15.24 | 1^st^: 76.50 ± 22.17 2^nd^: 81.00 ± 18.71 3^rd^: 96.60 ± 16.63 | 1^st^: +8.20 2^nd^: +12.70 3^rd^: +28.30 | 1^st^: 12.01 2^nd^: 18.59 3^rd^: 41.43 |
| Quary and Spodick (1974) [110] | Sitting 1^st^: 118.40 ± 16.29 2^nd^: 116.90 ± 11.76 3^rd^: 119.00 ± 13.60 4^th^: 117.60 ± 14.86 Supine 1^st^: 119.00 ± 10.56 2^nd^: 119.00 ± 15.08 3^rd^: 123.60 ± 14.26 4^th^: 125.30 ± 16.85 | Sitting 1^st^: 126.00 ± 21.35 2^nd^: 140.40 ± 23.37 3^rd^: 142.70 ± 24.16 4^th^: 165.20 ± 29.28 Supine 1^st^: 125.50 ± 15.65 2^nd^: 136.40 ± 12.78 3^rd^: 141.10 ± 14.55 4^th^: 169.10 ± 23.34 | Sitting 1^st^: +7.60 2^nd^: +23.50 3^rd^: +23.70 4^th^: +47.60 Supine 1^st^: +6.50 2^nd^: +17.40 3^rd^: +17.50 4^th^: +43.80 | Sitting 1^st^: 6.42 2^nd^: 20.10 3^rd^: 19.92 4^th^: 40.48 Supine 1^st^: 5.46 2^nd^: 14.62 3^rd^: 14.16 4^th^: 34.96 | Sitting 1^st^: 77.30 ± 11.64 2^nd^: 79.40 ± 14.67 3^rd^: 81.10 ± 10.25 4^th^: 87.80 ± 10.06 Supine 1^st^: 80.70 ± 12.78 2^nd^: 82.00 ± 10.91 3^rd^: 82.20 ± 8.79 4^th^: 83.80 ± 8.19 | Sitting 1^st^: 83.00 ± 14.70 2^nd^: 99.20 ± 19.89 3^rd^: 102.10 ± 12.87 4^th^: 114.30 ± 15.72 Supine 1^st^: 83.70 ± 9.52 2^nd^: 93.00 ± 10.02 3^rd^: 101.90 ± 10.06 4^th^: 125.10 ± 21.85 | Sitting 1^st^: +5.70 2^nd^: +19.80 3^rd^: +21.00 4^th^: +26.50 Supine 1^st^: +3.00 2^nd^: +11.00 3^rd^: +19.70 4^th^: +41.30 | Sitting 1^st^: 7.37 2^nd^: 24.94 3^rd^: 25.89 4^th^: 30.18 Supine 1^st^: 3.72 2^nd^: 13.41 3^rd^: 23.97 4^th^: 49.28 |
|  |  |  |  |  |  |  |  |  |
|  |  |  |  |  |  |  |  |  |
|  |  |  |  |  |  |  |  |  |
|  |  |  |  |  |  |  |  |  |
|  |  |  |  |  |  |  |  |  |
|  |  |  |  |  |  |  |  |  |
|  |  |  |  |  |  |  |  |  |
| Sagiv et al. (1985) [112] | 118.00 ± 8.00 | 162.00 ± 17.00 | +44.00 | 37.29 | 70.00 ± 6.00 | 97.00 ± 8.00 | +27.00 | 38.57 |
| Sagiv et al. (1988c) [115] | Young  118.60 ± 9.70 Elderly  118.60 ± 8.20 | Young  148.80 ± 15.80 Elderly  157.70 ± 13.60 | Young  +30.20 Elderly  +39.10 | Young  25.46 Elderly  32.97 | Young  72.80 ± 6.70 Elderly  70.00 ± 6.60 | Young  95.00 ± 9.40 Elderly  100.60 ± 12.10 | Young  +22.20 Elderly  +30.60 | Young  30.49 Elderly  43.71 |
| Sagiv et al. (1995) [116] | Direct measure  126.00 ± 17.00 Indirect measure  129.00 ± 16.00 | Direct measure  134.00 ± 18.00 Indirect measure  139.00 ± 18.00 | Direct measure  +8.00 Indirect measure  +10.00 | Direct measure  6.35 Indirect measure  7.75 | Direct measure  76.00 ± 12.00 Indirect measure  86.00 ± 10.00 | Direct measure  85.00 ± 12.00 Indirect measure  101.00 ± 9.00 | Direct measure  +9.00 Indirect measure  +15.00 | Direct measure  11.84 Indirect measure  17.44 |
| Samora et al. (2019) [118] | Men  114.00 ± 8.94 Women  98.00 ± 4.47 | Men  168.00 ± 22.36 Women  133.00 ± 17.89 | Men  +54.00 Women  +5.00 | Men  47.37 Women  35.71 | Men  60.00 ± 4.47 Women  56.00 ± 4.47 | Men  92.00 ± 13.42 Women  81.00 ± 8.94 | Men  +32.00 Women  +25.00 | Men  53.33 Women  44.64 |
|  |  |  |  |  |  |  |  |  |
| Seals  (1989) [119] | Right  147.00 ± 15.00  Left  147.00 ± 12.00 Bilateral  146.00 ± 12.00 | Right  175.00 ± 15.00 Left  171.00 ± 21.00 Bilateral  185.00 ± 21.00 | Right  +28.00 Left  +24.00 Bilateral  +39.00 | Right  19.05 Left  16.33 Bilateral  26.71 | NR | NR | NR | _ |
| Seals et al. (1985) [120] | Before  121.0 ± 10.0 After  111.0 ± 14.0 | Before 1^st^: 171.0 ± 18.0 2^nd^: 169.0 ± 26.0 After 1^st^: 153.0 ± 21.0 2^nd^: 152.0 ± 23.0 | Before 1^st^: + 50.00 2^nd^: + 48.00 After 1^st^: + 42.00 2^nd^: + 41.00 | Before 1^st^: 41.32 2^nd^: 39.67 After 1^st^: 37.84 2^nd^: 36.94 | Before  78.0 ± 12.0 After  68.0 ± 8.0 | Before 1^st^: 113.0 ± 15.0 2^nd^ :117 .0 ± 14.0 After 1^st^: 99.0 ± 12.0 2^nd^: 101.0 ± 13.0 | Before 1^st^: + 35.00 2^nd^: + 39.00 After 1^st^: + 31.00 2^nd^: + 33.00 | Before  1^st^: 44.87 2^nd^: 50.00 After  1^st^: 45.59 2^nd^: 48.53 |
|  |  |  |  |  |  |  |  |  |
|  |  |  |  |  |  |  |  |  |
|  |  |  |  |  |  |  |  |  |
|  |  |  |  |  |  |  |  |  |
|  |  |  |  |  |  |  |  |  |
| Somani et al. (2017) [28] | Men  117.00 ± 5.00 Women  103.00 ± 6.00 | NR | Men  +22.00 ± 8.00 Women  +14.00 ± 3.00 | Men  18.80 Women  13.59 | Men  65.00 ± 7.00 Women  62.00 ± 8.00 | NR | Men  +11.00 ± 5.00 Women  +9.00 ± 5.00 | Men  16.92 Women  14.52 |
| Stewart el al. (2006) [121] | 118.00 ± 10.00 | 148.00 ± 14.00 | +30.00 | 25.42 | NR | NR | NR | _ |
| Tan et al. (2013) [122] | 110.80 ± 6.96 | 160.30 ± 15.26 | +49.50 | 44.68 | 61.50 ± 7.63 | 95.80 ± 12.93 | +34.30 | 55.77 |
| Turley et al. (2005) [124] | Men  125.00 ± 6.00 Women  108.00 ± 9.00 | NR | Men  +29.00 ± 11.00 Women  +23.00 ± 10.00 | Men  23.20 Women  1.30 | Men  71.00 ± 6.00 Women  62.00 ± 7.00 | NR | Men  +29.00 ± 11.00 Women  +23.00 ± 10.00 | Men  40.85 Women  37.10 |
|  |  |  |  |  |  |  |  |  |
| Umeda et al. (2009) [125] | 114.87 ± 10.10 | 60 seconds  140.30 ± 15.03 180 seconds  157.22 ± 17.76 | 60 seconds  +25.43 180 seconds  +42.35 | 60 seconds  22.14 180 seconds  36.87 | 69.52 ± 11.08 | 60 seconds  90.52 ± 13.19 180 seconds  104.17 ± 14.55 | 60 seconds  +21.00 180 seconds +34.65 | 60 seconds  30.21  180 seconds  49.84 |
| Umeda et al. (2015) [126] | African Americans 116.36 ± 13.13 Non-Hispanic White 121.5 ± 12.27 | African Americans 136.21 ± 19.33 Non-Hispanic White 132.36 ± 10.03 | African Americans +19.85 Non-Hispanic White  +10.86 | African Americans 17.06 Non-Hispanic White  8.94 | African Americans 65.36 ± 12.54 Non-Hispanic White 70.71 ± 7.81 | African Americans 83.00 ± 12.82 Non-Hispanic White 78.79 ± 4.66 | African Americans +17.64 Non-Hispanic White +8.08 | African Americans 26.99 Non-Hispanic White  11.43 |
|  |  |  |  |  |  |  |  |  |
| Van Huysduynen et al. (2004) [127] | 122.00 ± 15.00 | 151.00 ± 17.00 | +29.00 | 23.77 | NR | NR | NR | _ |
| Vaz et al. (1993) [128] | 110.50 ± 6.70 | 133.70 ± 15.00 | +23.20 ± 12.90 | 21.00 | 67.00 ± 6.40 | 92.20 ± 6.70 | +25.20 ± 9.00 | 37.61 |
| Vianna et al. (2012) [129] | 135.00 ± 5.66 | 165.00 ± 14.14 | +30.00 | 22.22 | 78.00 ± 8.49 | 100.00 ± 8.49 | +22.00 | 28.21 |
| Williams (1991) [132] | 1^st^: 156.00 ± 17.15 2^nd^: 153.00 ± 14.70 | 1^st^: 233.00 ± 22.05 2^nd^: 255.00 ± 22.05 | 1^st^: +77.00 ± 17.15 2^nd^: +102.00 ± 14.70 | 1^st^: 49.36 2^nd^: 66.67 | 1^st^: 96.00 ± 9.80 2^nd^: 92.00 ± 7.35 | 1^st^: 162.00 ± 17.15 2^nd^: 169.00 ± 19.60 | 1^st^: +66.00 ± 14.70 2^nd^: +77.00 ± 12.25 | 1^st^: 68.75 2^nd^: 83.70 |

Note: Data presented as mean ± standard deviation. Δ: BP during exercise - BP pre-exercise. % Δ: percentage difference from BP pre-exercise. NT: normotensive. HT: hypertensive. HG1: handgrip with post-exercise ischemia. HG2: handgrip with post-exercise ischemia + cold pressure test. FH-: without family history of hypertension. FH+: with family history of hypertension. Women NC: not taking oral contraceptive. Women OC: taking oral contraceptives; NR: not reported.
