# Supplementary material for: Acute Blood Pressure Response to Different Types of Isometric Exercise: A Systematic Review with Meta-Analysis
Source: Rev Cardiovasc Med. 2023 Feb 10;24(2):60. doi: 10.31083/j.rcm2402060 (PMC11273125; doi:10.31083/j.rcm2402060)
Supplement: Supplementary file 1 [file 2153-8174-24-2-060-s1.zip › Supplementary material 4.docx]

| **Blood pressure responses during different types of upper limb isometric exercise.** | | | | | | | | |
| --- | --- | --- | --- | --- | --- | --- | --- | --- |
| **Author and year** | **Pre SBP** | **During SBP** | **Δ SBP** | **Mean %Δ** | **Pre DBP** | **During DBP** | **Δ DBP** | **Mean %Δ** |
| ***Elbow flexion*** | | | | | | | | |
| Davies and Starkie  (1985) [49] | 125.00 ± 14.00 | 153.00 ± 15.00 | +28.00 | 22.40 | 80.00 ± 11.00 | 101.00 ± 12.00 | +21.00 | 26.25 |
| Mortensen et al. (2016) [97] | 117.00 ± 10.30 | 143.20 ± 18.30 | +26.20 | 22.39 | 69.50 ± 7.00 | 86.70 ± 9.70 | +17.20 | 24.75 |
| Nakamura et al. (2005) [100] | 141.10 ± 6.22 | 185.00 ± 31.11 | +43.90 | 31.11 | 85.50 ± 9.62 | 116.00 ± 26.59 | +30.50 | 35.67 |
| Yamaji et al.  (1983) [25] | 1^st^: 127.40 ± 10.29 2^nd^: 123.60 ± 11.24 3^rd^: 124.20 ± 10.38 4^th^: 125.70 ± 10.21 5^th^: 126.50 ± 10.47 | 1^st^: 140.30 ± 15.40 2^nd^: 148.50 ± 18.40 3^rd^: 159.30 ± 17.32 4^th^: 166.40 ± 15.49 5^th^: 168.60 ± 15.05 | 1^st^: +12.90 2^nd^: +24.90 3^rd^: +35.10 4^th^: +40.70 5^th^: +42.10 | 1^st^: 10.13 2^nd^: 20.15 3^rd^: 28.26 4^th^: 32.38 5^th^: 33.28 | 1^st^: 79.40 ± 6.77 2^nd^: 77.00 ± 9.02 3^rd^: 77.80 ± 6.22 4^th^: 77.20 ± 7.96 5^th^: 77.10 ± 7.19 | 1^st^: 88.30 ± 12.35 2^nd^: 97.10 ± 10.81 3^rd^: 103.70 ± 12.62 4^th^: 107.80 ± 12.71 5^th^: 107.60 ± 12.92 | 1^st^: +8.90 2^nd^: +20.10 3^rd^: +25.90 4^th^: +30.60 5^th^: +30.50 | 1^st^: 11.21 2^nd^: 26.10 3^rd^: 33.29 4^th^: 39.64 5^th^: 39.56 |
| ***Elbow extension*** | | | | | | | | |
| Seals et al.  (1983) [23] | Untrained member  122.00 ± 6.00 Trained member  122.00 ± 4.00 | Untrained member  158.00 ± 16.00 Trained member  163.00 ± 15.00 | Untrained member  +36.00 Trained member +41.00 | Untrained member  29.51 Trained member  33.61 | Untrained member  84.00 ± 9.00 Trained member  81.00 ± 9.00 | Untrained member  113.00 ± 14.00  Trained member  12.00 ± 14.00 | Untrained member  +29.00 Trained member +31.00 | Untrained member  34.52 Trained member TN 38.27 |
| ***Two-hand pulling*** | | | | | | | | |
| Ben-Ari et al.  (1992) [45] | 122.00 ± 15.00 | 150.00 ± 17.00 | +28.00 | 22.95 | 78.00 ± 5.00 | 85.00 ± 4.00 | +7.00 | 8.97 |
| ***Finger adduction*** | | | | | | | | |
| Riendl et al.  (1977) [111] | 2^nd^: 122.00 ± 10.12 | NR | 2^nd^: +35.00 ± 8.85 | 2^nd^: 28.69 | 2^nd^: 74.20 ± 5.69 | NR | 2^nd^: +32.30 ± 10.75 | 2^nd^: 43.53 |
| Note: Data presented as mean ± standard deviation. Δ= BP during exercise - BP pre-exercise. % Δ= percentage difference from BP pre-exercise. NR= not reported. | | | | | | | | |
