# Supplementary material for: Acute Blood Pressure Response to Different Types of Isometric Exercise: A Systematic Review with Meta-Analysis
Source: Rev Cardiovasc Med. 2023 Feb 10;24(2):60. doi: 10.31083/j.rcm2402060 (PMC11273125; doi:10.31083/j.rcm2402060)
Supplement: Supplementary file 1 [file 2153-8174-24-2-060-s1.zip › Supplementary material 5.docx]

| **Blood pressure responses during different types of lower limb isometric exercise.** | | | | | | | | |
| --- | --- | --- | --- | --- | --- | --- | --- | --- |
| **Author and year** | **Pre SBP** | **During SBP** | **Δ SBP** | **Mean %Δ** | **Pre DBP** | **During DBP** | **Δ DBP** | **Mean %Δ** |
| ***One-knee extension*** | | | | | | | | |
| Fujisawa et al.  (1996) [56] | 113.00 ± 15.00 | 191.00 ± 22.00 | +78.00 | 69.03 | 66.00 ± 8.00 | 129.00 ± 20.00 | +63.00 | 95.45 |
| Hirasawa et al. (2016) [70] | 116.00 ± 11.00 | 150.00 ± 16.00 | +34.00 | 29.31 | 64.00 ± 9.00 | 87.00 ± 10.00 | +23.00 | 35.94 |
| Iellamo et al.  (1999) [74] | 118.20 ± 9.62 | 144.70 ± 18.90 | +26.50 | 22.42 | 74.80 ± 8.95 | 90.30 ± 11.94 | +15.50 | 20.72 |
| Kadetoff and Kosek (2007) [76] | 114.88 ± 12.32 | 144.13 ± 12.32 | +29.25 | 25.46 | 76.06 ± 6.04 | 95.06 ± 8.53 | +19.00 | 24.98 |
| Seals et al.  (1983) [23] | Untrained  122.00 ± 6.00 Trained  122.00 ± 4.00 | Untrained  164.00 ± 15.00 Trained  164.00 ± 9.00 | Untrained  +42.00 Trained  +42.00 | Untrained  34.43 Trained  34.43 | Untrained  84.00 ± 9.00 Trained  81.00 ± 9.00 | Untrained  117.00 ± 13.00 Trained  121.00 ± 9.00 | Untrained  +33.00 Trained  +40.00 | Untrained  39.29 Trained  49.38 |
| Wright et al.  (1999) [133] | African American men  124.00 ± 10.46 African American women  111.00 ± 10.46  Asian American men  114.00 ± 4.26 Asian American women  101.00 ± 5.81  Caucasian American men  121.00 ± 7.36 Caucasian American women  111.00 ± 7.36 | African American men  159.00 ± 14.33  African American women  147.00 ± 10.84  Asian American men  149.00 ± 15.49  Asian American women  139.00 ± 8.52  Caucasian American men  162.00 ± 20.91 Caucasian American women  137.00 ± 12.78 | African American men  +35.00 African American women  +36.00  Asian American men  +35.00 Asian American women  +38.00  Caucasian American men  +41.00 Caucasian American women  +26.00 | African American men  28.23 African American women  32.43  Asian American men  30.70 Asian American women  37.62  Caucasian American men  33.88 Caucasian American women  23.42 | African American men  74.00 ± 8.13 African American women  74.00 ± 6.97  Asian American men  74.00 ± 5.42 Asian American women  70.00 ± 5.42  Caucasian American men  75.00 ± 7.36 Caucasian American women  71.00 ± 6.20 | African American men  107.00 ± 13.56 African American women  103.00 ± 17.04  Asian American men  102.00 ± 14.72 Asian American women  98.00 ± 14.33  Caucasian American men  107.00 ± 12.78 Caucasian American women  99.00 ± 14.72 | African American men  +33.00 African American women  +29.00  Asian American men  +28.00 Asian American women  +28.00  Caucasian American men  +32.00 Caucasian American women  +28.00 | African American men  44.59 African American women  39.19  Asian American men  37.84 Asian American women  40.0  Caucasian American men  42.67  Caucasian American women  39.44 |
| Yamaji et al.  (1983) [25] | 1^st^: 123.60 ± 8.66 2^nd^: 122.60 ± 6.45 3^rd^: 122.40 ± 8.05 4^th^: 125.20 ± 8.27 5^th^: 124.00 ± 6.94 | 1^st^: 141.70 ± 15.24 2^nd^: 147.70 ± 14.56 3^rd^: 158.20 ± 23.01 4^th^: 154.20 ± 16.19 5^th^: 154.80 ± 14.71 | 1^st^: +18.10 2^nd^: +25.10 3^rd^: +35.80 4^th^: +29.00 5^th^: +30.80 | 1^st^: 14.64 2^nd^: 20.47 3^rd^: 29.25 4^th^: 23.17 5^th^: 24.84 | 1^st^: 76.50 ± 7.79 2^nd^: 77.30 ± 6.89 3^rd^: 79.10 ± 7.76 4^th^: 79.20 ± 8.00 5^th^: 78.00 ± 7.90 | 1^st^: 90.80 ± 10.92 2^nd^: 95.00 ± 11.81 3^rd^: 100.20 ± 12.08 4^th^: 100.40 ± 8.57 5^th^: 99.60 ± 9.57 | 1^st^: +14.30 2^nd^: +17.70 3^rd^: +21.10 4^th^: +21.20 5^th^: +21.60 | 1^st^: 18.69 2^nd^: 22.90 3^rd^: 26.68 4^th^: 26.77 5^th^: 27.69 |
| ***Two-knee extension*** | | | | | | | | |
| Hickey et al.  (1993) [69] | Morning  114.76 ± 7.50 Afternoon  122.26 ± 6.75 | Morning  190.52 ± 15.75 Afternoon  201.02 ± 8.25 | Morning  +75.76 Afternoon  +78.76 | Morning  66.02 Afternoon  64.42 | Morning  74.25 ± 5.25 Afternoon  72.76 ± 6.75 | Morning  125.26 ± 4.50 Afternoon  130.51 ± 8.25 | Morning  +51.01 Afternoon  +57.75 | Morning  68.70 Afternoon  79.37 |
|  |  |  |  |  |  |  |  |  |
| Kadetoff and Kosek (2010) [76] | 115.00 ± 12.00 | 144.00 ± 12.00 | +29.00 | 25.22 | 76.00 ± 6.00 | 95.00 ± 8.00 | +19.00 | 25.00 |
| Lewis et al.  (1985) [24] | 118.00 ± 7.36 | 193.00 ± 17.15 | +75.00 | 63.56 | 67.00 ± 7.35 | 114.00 ± 4.90 | +47.00 | 70.15 |
| Nagle et al.  (1988) [99] | 123.00 ± 8.00 | 157.00 ± 11.00 | +34.00 | 27.64 | 79.00 ± 4.00 | 110.00 ± 8.00 | +31.00 | 39.24 |
| Petrosfsky and Laymon  (2002) [107] | 20-30 years  120.76 ± 9.00 31-40 years  123.01 ± 8.30 41-50 years  129.01 ± 9.00 51-65 years  135.01 ± 8.30 | 20-30 years  185.27 ± 12.80 31-40 years  186.77 ± 9.80 41-50 years  191.27 ± 11.30 51-65 years  201.01 ± 15.00 | 20-30 years  +64.51 31-40 years  +63.76 41-50 years  +62.26 51-65 years  +66.00 | 20-30 years  53.42 31-40 years  51.83 41-50 years  48.26 51-65 years  48.89 | 20-30 years  78.00 ± 11.30 31-40 years  76.50 ± 9.00 41-50 years  83.25 ± 12.00 51-65 years  84.75 ± 8.30 | 20-30 years  134.26 ± 12.80 31-40 years  138.01 ± 11.30 41-50 years  141.76 ± 12.00 51-65 years  147.01 ± 15.80 | 20-30 years  +56.26 31-40 years  +61.51 41-50 years  +58.51 51-65 years  +62.26 | 20-30 years  72.13 31-40 years  80.41 41-50 years  70.28 51-65 years  73.46 |
| Somani et al.  (2017) [28] | Men  126.00 ± 8.00 Women  122.00 ± 5.00 | NR | Men  +35.00 ± 17.00 Women  +25.00 ± 13.00 | Men  27.78 Women  20.49 | Men  73.00 ± 5.00 Women  73.00 ± 5.00 | NR | Men  +27.00 ± 12.00 Women  +21.00 ± 16.00 | Men  36.99 Women  28.77 |
| Williams  (1991) [132] | 1^st^: 138.00 ± 41.64 2^nd^: 133.00 ± 26.94 | 1^st^: 224.00 ± 51.44 2^nd^: 260.00 ± 71.04 | 1^st^: +86.00 ± 9.80 2^nd^: +127.00 ± 95.53 | 1^st^: 62.32 2^nd^: 95.49 | 1^st^: 82.00 ± 29.39 2^nd^: 80.00 ± 24.49 | 1^st^: 156.00 ± 63.69 2^nd^: 178.00 ± 58.79 | 1^st^: +74.00 ± 34.29 2^nd^: +98.00 ± 46.54 | 1^st^: 90.24 2^nd^: 122.5 |
| ***Squat*** | | | | | | | | |
| Dias and Polito  (2015) [50] | 127.10 ± 6.10 | 193.20 ± 12.90 | +66.10 | 52.01 | 77.40 ± 3.80 | 127.40 ± 11.60 | +50.00 | 64.60 |
| Taylor et al.  (2017) [123] | 132.60 ± 5.60 | 165.90 ± 21.00 | +33.30 | 25.11 | 78.00 ± 9.00 | NR | NR | _ |
| Wiles et al.  (2018) [18] | 132.00 ± 6.00 | 171.00 ± 19.00 | +39.00 | 29.55 | 76.00 ± 8.00 | 113.00 ± 11.00 | +37.00 | 48.68 |
| ***Leg press*** | | | | | | | | |
| Da Silva et al.  (2013) [26] | High intensity 119.00 ± 14.37 Moderate intensity  122.88 ± 13.43 Low intensity 113.13 ± 10.67 | High intensity 195.00 ± 22.80 Moderate intensity  181.00 ± 20.20 Low intensity 158.10 ± 20.20 | High intensity  +76.00 Moderate intensity  +58.12 Low intensity +44.97 | High intensity  63.87 Moderate intensity  47.30 Low intensity  39.75 | High intensity  74.88 ± 12.96 Moderate intensity  64.38 ± 12.37 Low intensity  66.25 ± 10.94 | High intensity 119.40 ± 21.10 Moderate intensity  110.60 ± 17.40 Low intensity  96.30 ± 20.50 | High intensity +44.52 Moderate intensity  +46.22 Low intensity +30.05 | High intensity 59.46 Moderate intensity  71.79 Low intensity 45.36 |
| Weippert et al. (2013) [131] | 129.00 ± 8.60 | 158.00 ± 12.10 | +29.00 | 22.48 | 76.50 ± 7.10 | 95.30 ± 6.30 | +18.80 | 24.54 |
| ***Plantar flexion*** | | | | | | | | |
| Davies and Starkie (1985) [49] | 1^st^:127.00 ± 10.00 2^nd^: 125.00 ± 12.00 | 1^st^: 154.00 ± 13.00 2^nd^: 144.00 ± 15.00 | 1^st^: +27.00 2^nd^: +19.00 | 1^st^: 21.26 2^nd^: 15.20 | 1^st^: 82.00 ± 10.00 2^nd^: 81.00 ± 6.00 | 1^st^: 106.00 ± 8.00 2^nd^: 102.00 ± 10.00 | 1^st^: +24.00 2^nd^: +21.00 | 1^st^: 29.27 2^nd^: 25.93 |
| Riendl et al.  (1977) [111] | 1^st^: 122.60 ± 11.07 | NR | 1^st^: +33.40 ± 17.08 | 27.24 | 1^st^: 80.50 ± 5.06 | NR | 1^st^: +23.00 ± 12.33 | 28.57 |
| Note: Data presented as mean ± standard deviation. Δ: BP during exercise - BP pre-exercise. % Δ: percentage difference from BP pre-exercise. NR: not reported. | | | | | | | | |
