# Supplementary material for: Acute Blood Pressure Response to Different Types of Isometric Exercise: A Systematic Review with Meta-Analysis
Source: Rev Cardiovasc Med. 2023 Feb 10;24(2):60. doi: 10.31083/j.rcm2402060 (PMC11273125; doi:10.31083/j.rcm2402060)
Supplement: Supplementary file 1 [file 2153-8174-24-2-060-s1.zip › Supplementary material 6.docx]

| **Blood pressure responses during different types of isometric exercise involving the trunk or the whole body.** | | | | | | | | |
| --- | --- | --- | --- | --- | --- | --- | --- | --- |
| **Author and year** | **Pre SBP** | **During SBP** | **Δ SBP** | **Mean %Δ** | **Pre DBP** | **During DBP** | **Δ DBP** | **Mean %Δ** |
|  |  |  |  | ***Deadlift*** |  |  |  |  |
| Nagle et al.  (1988) [99] | 123.00 ± 8.00 | 168.00 ± 23.00 | +45.00 | 36.59 | 79.00 ± 4.00 | 114.00 ± 12.00 | +35.00 | 44.30 |
| Sagiv et al.  (1985) [112] | 118.00 ± 8.00 | 189.00 ± 26.00 | +71.00 | 60.17 | 70.00 ± 6.00 | 105.00 ± 14.00 | +35.00 | 50.00 |
| Sagiv et al.  (1988) [113] | Young  119.00 ± 10.00 Elderly  119.00 ± 8.00 | Young  179.00 ± 19.00  Elderly  182.00 ± 22.00 | Young  +60.00 Elderly  +63.00 | Young  50.42 Elderly  52.94 | Young  73.00 ± 7.00  Elderly  70.00 ± 7.00 | Young  108.00 ± 11.00  Elderly  106.00 ± 13.00 | Young  +35.00 Elderly  +36.00 | Young  47.95 Elderly  51.43 |
|  |  |  |  |  |  |  |  |  |
| Sagiv et al. (1988b) [114] | Young  118.60 ± 9.70  Middle-aged  118.60 ± 8.20  Elderly  120.50 ± 11.80 | Young  178.60 ± 18.00  Middle-aged  181.80 ± 22.00  Elderly  194.30 ± 26.00 | Young  +60.00  Middle-aged  +63.20  Elderly  +73.80 | Young  50.59  Middle-aged  53.29  Elderly  61.24 | Young  72.80 ± 6.70  Middle-aged  70.00 ± 6.60  Elderly  73.60 ± 9.10 | Young  107.90 ± 10.60  Middle-aged  105.60 ± 12.80  Elderly  106.30 ± 16.10 | Young  +35.10  Middle-aged  +35.60  Elderly  +32.70 | Young  48.21  Middle-aged  50.86  Elderly  44.43 |
|  |  |  |  |  |  |  |  |  |
|  |  |  |  |  |  |  |  |  |
| Sagiv et al. (1988c) [115] | Young  118.60 ± 9.70 Elderly  118.60 ± 8.20 | Young  178.60 ± 19.00 Elderly  181.80 ± 22.40 | Young  +60.00 Elderly  +63.20 | Young  50.59 Elderly  53.29 | Young  72.80 ± 6.70 Elderly  70.00 ± 6.60 | Young  107.90 ± 10.60 Elderly  105.60 ± 12.80 | Young  +35.10 Elderly  +35.60 | Young  48.21 Elderly  50.86 |
| Sagiv et al.  (1995) [116] | Direct measure 126.00 ± 17.00 Indirect measure 129.00 ± 16.00 | Direct measure  162.00 ± 27.00 Indirect measure 167.00 ± 25.00 | Direct measure  +36.00 Indirect measure +38.00 | Direct measure  28.57 Indirect measure 29.46 | Direct measure  76.00 ± 12.00 Indirect measure  86.00 ± 10.00 | Direct measure  100.00 ± 14.00 Indirect measure  110.00 ± 11.00 | Direct measure  +24.00 Indirect measure  +24.00 | Direct measure 31.58 Indirect measure 27.91 |
| Sagiv et al.  (2008) [117] | 116.90 ± 7.30 | 188.10 ± 30.00 | +71.20 | 60.91 | 72.90 ± 8.00 | 106.90 ± 10.50 | +34.00 | 46.64 |
| Vitcenda et al. (1990) [130] | 142.00 ± 21.00 | 217.00 ± 22.00 | +75.00 | 52.82 | 89.00 ± 13.00 | 150.00 ± 27.00 | +61.00 | 68.54 |
| ***Whole-body isometric exercise*** | | | | | | | | |
| Auerbach et al. (2000) [41] | 125.50 ± 9.70 | 1^st^: NR  2^nd^: 165.50 ± 8.50 | 1^st^: NC  2^nd^: +40.00 | 1^st^: NR 2^nd^: 31.87 | 81.90 ± 6.50 | 1^st^: NR  2^nd^: 100.50 ± 4.50 | 1^st^: NC 2^nd^: +18.60 | 1^st^: NR 2^nd^: 22.71 |
| ***Torso effort*** | | | | | | | | |
| Plotnikov et al. (2002) [109] | 106.20 ± 15.24 | 1^st^: 114.00 ± 24.25 2^nd^: 129.80 ± 24.94 3^rd^: 137.10 ± 19.40 | 1^st^: +7.80 2^nd^: +23.60 3^rd^: +30.90 | 1^st^: 7.34 2^nd^: 22.22 3^rd^: 29.10 | 70.20 ± 10.39 | 1^st^: 87.30 ± 22.17 2^nd^: 95.90 ± 17.32 3^rd^: 98.90 ± 18.71 | 1^st^: +17.10 2^nd^: +25.70 3^rd^: +28.70 | 1^st^: 24.36 2^nd^: 36.61 3^rd^: 40.88 |

Note: Data presented as mean ± standard deviation. Δ: BP during exercise – BP pre-exercise. % Δ: percentage difference from BP pre-exercise. NR: not reported. NC: not calculated.
