# Supplementary material for: Acute Blood Pressure Response to Different Types of Isometric Exercise: A Systematic Review with Meta-Analysis
Source: Rev Cardiovasc Med. 2023 Feb 10;24(2):60. doi: 10.31083/j.rcm2402060 (PMC11273125; doi:10.31083/j.rcm2402060)
Supplement: Supplementary file 1 [file 2153-8174-24-2-060-s1.zip › Supplementary material 7.docx]

| Blood pressure response when comparing isometric exercise types. | | | | | | |
| --- | --- | --- | --- | --- | --- | --- |
| **Comparision of exercise types** | **Mean difference** | **Standard error** | **Variance** | **CI 95%** | **Z-value** | **P-value** |
| **PAS (mmHg)** |  | | | | | |
| Handgrip x Elbow flexion | - 1.580 | 5.052 | 25.524 | -11.482; 8.322 | -0.313 | 0.754 |
| Handgrip x One-knee extension | + 2.080 | 5.147 | 26.493 | -8.008; 12.168 | 0.404 | 0.686 |
| Handgrip x Two-knee extension | + 36.080 | 6.663 | 44.400 | 23.020; 49.140 | 5.415 | <0.001 |
| Handgrip x Squat | +12.820 | 5.022 | 25.219 | 2.977; 22.663 | 2.553 | 0.011 |
| Handgrip x Leg Press | +18.720 | 6.034 | 36.414 | 6.893; 30.547 | 3.102 | 0.002 |
| Handgrip x Plantar flexion | -10.310 | 5.542 | 30.711 | -21.172; 0.552 | -1.860 | 0.063 |
| Handgrip x Deadlift | +26.640 | 6.521 | 42.527 | 13.859; 39.421 | 4.085 | <0.001 |
| Handgrip x Torso effort | -12.540 | 2.233 | 4.985 | -16.916; -8.164 | -5.616 | <0.001 |
| Elbow flexion x One-knee extension | +3.660 | 4.947 | 24.475 | -6.036; 13.356 | 0.740 | 0.459 |
| Elbow flexion x Two-knee extension | +37.660 | 6.265 | 39.256 | 25.380; 49.940 | 6.011 | <0.001 |
| Elbow flexion x Squat | +14.400 | 4.555 | 20.751 | 5.472; 23.328 | 3.161 | 0.002 |
| Elbow flexion x Leg press | +20.300 | 5.708 | 32.587 | 9.112; 31.488 | 3.556 | <0.001 |
| Elbow flexion x Plantar flexion | -8.730 | 5.453 | 29.732 | -19.417; 1.957 | -1.601 | 0.109 |
| Elbow flexion x Deadlift | +28.220 | 5.923 | 35.081 | 16.611; 39.829 | 4.765 | <0.001 |
| Elbow flexion x Torso effort | -10.960 | 2.350 | 5.522 | -15.566; -6.354 | -4.664 | <0.001 |
| One knee extension x Two-knee extension | +34.000 | 6.398 | 40.930 | 21.461; 46.539 | 5.314 | <0.001 |
| One knee extension x Squat | + 10.740 | 4.909 | 24.101 | 1.118; 20.362 | 2.188 | 0.029 |
| ­One knee extension x Leg press | + 16.640 | 5.780 | 33.410 | 5.311; 27.969 | 2.879 | 0.004 |
| One-knee extension x Plantar flexion | -12.390 | 5.206 | 27.103 | -22.594; -2.186 | -2.380 | 0.017 |
| One-knee extension x Deadlift | +24.560 | 6.360 | 40.453 | 12.094; 37.026 | 3.861 | <0.001 |
| One-knee extension x Torso effort | -14.620 | 2.026 | 4.106 | -18.592; -10.648 | -7.215 | <0,001 |
| Two-knee extension x Squat | -23.260 | 6.229 | 38.795 | -35.460; -11.052 | -3.734 | <0.001 |
| Two-knee extension x Leg press | -17.360 | 7.518 | 56.525 | -32.096; -2.624 | -2.309 | 0.021 |
| Two-knee extension x Plantar flexion | -46.390 | 6.903 | 47.653 | -59.920; -32.860 | -6.720 | <0.001 |
| Two-knee extension x Deadlift | -9.440 | 8.121 | 65.947 | -25.356; 6.476 | -1.162 | 0.245 |
| Two-knee extension x Torso effort | -48.620 | 2.752 | 7.575 | -54.014; -43.226 | -17.665 | <0.001 |
| Squat x Leg press | +5.900 | 5.671 | 32.161 | -5.215; 17.015 | 1.040 | 0.298 |
| Squat x Plantar flexion | -23.130 | 5.391 | 29.068 | -33.697; -12.563 | -4.290 | <0.001 |
| Squat x Deadlift | +13.820 | 5.915 | 34.989 | 2.227; 25.413 | 2.336 | 0.019 |
| Squat x Torso effort | -25.360 | 2.307 | 5.323 | -29.882; -20.838 | -10.992 | <0.001 |
| Leg press x Plantar flexion | -29.030 | 6.211 | 38.582 | -41.204; -16.856 | -4.674 | <0.001 |
| Leg press x Deadlift | +7.920 | 7.378 | 54.441 | -6.541; 22.381 | 1.073 | 0.283 |
| Leg press x Torso effort | -31.260 | 2.467 | 6.084 | -36.094; -26.426 | -12.674 | <0.001 |
| Plantar flexion x Deadlift | +36.950 | 6.992 | 48.888 | 23.246; 50.654 | 5.285 | <0.001 |
| Plantar flexion x Torso effort | -2,230 | 1.994 | 3.976 | -6.138; 1.678 | -1.118 | 0.263 |
| Deadlift x Torso effort | -39.180 | 2.946 | 8.677 | -44.953; -33.407 | -13.301 | <0.001 |
| **PAD (mmHg)** | | | | | | |
| Handgrip x Elbow flexion | -1.55 | 3.696 | 13.661 | -8.794 – 5.694 | -0.419 | 0.675 |
| Handgrip x One-knee extension | +2.170 | 3.864 | 14.927 | -5.403; 9.743 | 0.562 | 0.574 |
| Handgrip x Two-knee extension | +31.410 | 5.274 | 27.818 | 21.073; 41.747 | 5.955 | <0.001 |
| Handgrip x Squat | +17.750 | 3.295 | 10.858 | 11.291; 24.209 | 5.387 | <0.001 |
| Handgrip x Leg Press | +9.580 | 4,731 | 22.386 | 0.307; 18.853 | 2.025 | 0.043 |
| Handgrip x Plantar flexion | -2.820 | 3.789 | 14.354 | -10.246; 4.606 | -0.744 | 0.457 |
| Handgrip x Deadlift | +9.920 | 4.092 | 16.748 | 1,899; 17.941 | 2.424 | 0.015 |
| Handgrip x Torso effort | -1.490 | 1.582 | 2.502 | -4.590;1.610 | -0.942 | 0.346 |
| Elbow flexion x One-knee extension | +3.720 | 3.827 | 14.645 | -3.781; 11.221 | 0.972 | 0.331 |
| Elbow flexion x Two-knee extension | +32.960 | 5.011 | 25.110 | 23.139; 42.781 | 6.578 | <0.001 |
| Elbow flexion x Squat | +19.300 | 3.182 | 10.127 | 13.063; 25.537 | 6.065 | <0.001 |
| Elbow flexion x Leg press | +11.130 | 4.537 | 20.589 | 2.237; 20.023 | 2.453 | 0.014 |
| Elbow flexion x Plantar flexion | -1.270 | 4.009 | 16.071 | -9.127; 6.587 | -0.317 | 0.751 |
| Elbow flexion x Deadlift | +11.470 | 4.027 | 16.214 | 3.578; 19.362 | 2.849 | 0.004 |
| Elbow flexion x Torso effort | +0.060 | 1.781 | 3.172 | -3.431; 3.551 | 0.034 | 0.973 |
| One-knee extension x Two-knee extension | +29.240 | 5.479 | 30.024 | 18.501; 39.979 | 5.336 | <0.001 |
| One-knee extension x Squat | +15.580 | 3.424 | 11.726 | 8.868; 22.292 | 4.550 | <0.001 |
| One-knee extension x Leg press | +7.410 | 4.918 | 24.190 | -2.230; 17.050 | 1.507 | 0.132 |
| One-knee extension x Plantar flexion | -4.990 | 3.978 | 15.827 | -12.787; 2.807 | -1.254 | 0.210 |
| One-knee extension x Deadlift | +7.750 | 4.263 | 18.177 | -0.606; 16.106 | 1.818 | 0.069 |
| One-knee extension x Torso effort | -3.660 | 1.670 | 2.789 | -6.933; -0.387 | -2.192 | 0.028 |
| Two-knee extension x Squat | -13.660 | 4.577 | 20.953 | -22.632; -4.688 | -2.984 | 0.003 |
| Two-knee extension x Leg press | -21.830 | 6.599 | 43.553 | -34.765; -8.895 | -3.308 | 0.001 |
| Two-knee extension x Plantar flexion | -34.230 | 5.626 | 31.651 | -45.257; -23.203 | -6.084 | <0.001 |
| Two-knee extension x Deadlift | -21.490 | 5.786 | 33.482 | -32.831; -10.149 | -3.714 | <0.001 |
| Two-knee extension x Torso effort | -32.900 | 2.412 | 5.819 | -37.628; -28.172 | -13.639 | <0.001 |
| Squat x Leg press | -8.170 | 4.127 | 17.029 | -16.258; -0.082 | -1.980 | 0.048 |
| Squat x Plantar flexion | -20.570 | 3.467 | 12.020 | -27.365; -13.775 | -5.933 | <0.001 |
| Squat x Deadlift | -7.830 | 3.615 | 13.065 | -14.915; -0.745 | -2.166 | 0.030 |
| Squat x Torso effort | -19.240 | 1.501 | 2.253 | -22.182; -16.298 | -12.819 | <0.001 |
| Leg press x Plantar flexion | -12.400 | 5.008 | 25.083 | -22.216; -2.584 | -2.476 | 0.013 |
| Leg press x Deadlift | +0.340 | 5.196 | 26.995 | -9.843; 10.523 | 0.065 | 0.948 |
| Leg press x Torso effort | -11.070 | 2.143 | 4.593 | -15.270; -6.870 | -5.165 | <0.001 |
| Plantar flexion x Deadlift | +12.740 | 4.239 | 17.969 | 4.432; 21.048 | 3.005 | 0.003 |
| Plantar flexion x Torso effort | +1.330 | 1.405 | 1.974 | -1.424; 4.084 | 0.947 | 0.344 |
| Deadlift x Torso effort | -11.410 | 1.786 | 3.189 | -14.910; -7.910 | -6.390 | <0.001 |

Note: for the mean difference it was considered the difference of the second exercise type in relation to the first exercise type.
